# Generating human blastoids modeling blastocyst-stage embryos and implantation

**DOI:** 10.1038/s41596-023-00802-1

**Published:** 2023-02-15

**Authors:** Heidar Heidari Khoei, Alok Javali, Harunobu Kagawa, Theresa Maria Sommer, Giovanni Sestini, Laurent David, Jana Slovakova, Maria Novatchkova, Yvonne Scholte op Reimer, Nicolas Rivron

**Affiliations:** 1 Institute of Molecular Biotechnology of the Austrian Academy of Sciences (IMBA), Vienna BioCenter (VBC), Vienna, Austria; 2 Université de Nantes, CHU Nantes, Inserm, CR2TI, UMR 1064, F-44000, Nantes, France; 3 Université de Nantes, CHU Nantes, Inserm, CNRS, BioCore, F-44000, Nantes, France; 4 Institute of Molecular Biotechnology of the Austrian Academy of Science (IMBA), IMBA Stem Cell Core Facility (ISCCF), Vienna BioCenter (VBC), Vienna, Austria; 5 Institute of Molecular Pathology (IMP), Vienna Biocenter (VBC), Vienna, Austria

## Abstract

Human early development sets the stage for embryonic and adult life but remains difficult to investigate. A solution came from stem cells organizing into structures resembling pre-implantation embryos - blastocysts - that we termed blastoids. This embryo model is available in unlimited numbers and could thus support scientific and medical advances. However, its predictive power depends on how faithfully it recapitulates the blastocyst. Here, we describe how we formed human blastoids that (i) efficiently achieve the morphology of the blastocyst, (ii) form lineages according to the pace and sequence of blastocyst development, (iii) ultimately forming cells that transcriptionally reflect the blastocyst (pre-implantation stage). We employ three different commercially available 96- and 24-well microwell plates with results similar to our custom-made ones, and show that blastoids form in clinical IVF media and can be cryopreserved for shipping. Finally, we explain how blastoids replicate the directional process of implantation into endometrial organoids, specifically when these are hormonally stimulated. It takes 4 days for human blastoids to form, 10 days to prepare the endometrial implantation assay, and we have cultured blastoids up to 6 days (time-equivalent of day 13). Based on our experience, we anticipate that a person with about 1 year of human Pluripotent Stem Cell (hPSC) culture experience and organoid culture should be able to perform the protocol. Altogether, blastoids offer an opportunity to establish scientific and biomedical discovery programs for early pregnancy, and an ethical alternative to the use of embryos.

## Introduction

### Development of the protocol

Through *in vitro* fertilization (IVF), human embryos can form and develop in a dish for 5-6 days, which makes them visible and accessible for research. However, such embryos are scarce and remain difficult to manipulate^1,2^, which hinders our understanding of their development and our possibilities to optimize their extended culture^3–5^. During IVF procedures, day 5-6 embryos are transferred in the womb, become inaccessible, and can only be rudimentarily observed again, ultrasonographically, after ∼5 weeks. Interestingly, this period of human implantation and early development is especially prone to failure as early abnormalities or insults result in infertility, pregnancy failure, and contribute to the developmental origin of health and diseases^6–11^.

Important progress in our understanding of early mammalian development was made using model organisms, mostly mice^12^. Although more readily available than human ones, mouse embryos are also not abundant enough to be amenable to screening (genetic and pharmacological), nor to complicated alterations (e.g., complex genetic editing, precise mix of genotypes). Also, species differences restrict a direct homology to the human case. Most notably, mouse blastocysts implant *in utero via* the opposite side as compared to human ones^13^.

Consequently, there is a need for a scalable model of the human blastocyst allowing for high-throughput and complex mechanistic studies. Altogether, this sparked the idea of forming embryo models from stem cells as a technical but also ethical alternative to the use of embryos for research^14^. Initially, a model of the mouse blastocyst, called a blastoid, was formed that morphologically and transcriptionally resembled the blastocyst, generated the 3 founding lineages (trophectoderm (TE), epiblast (EPI), and primitive endoderm (PrE)), and implanted *in utero*^15^. However, it is only recently that advances in hPSCs culture conditions have exposed the ability to form analogs of the blastocyst-stage TE^16–22^. This opened the possibility to form human blastoids.

**Fig. 1:**
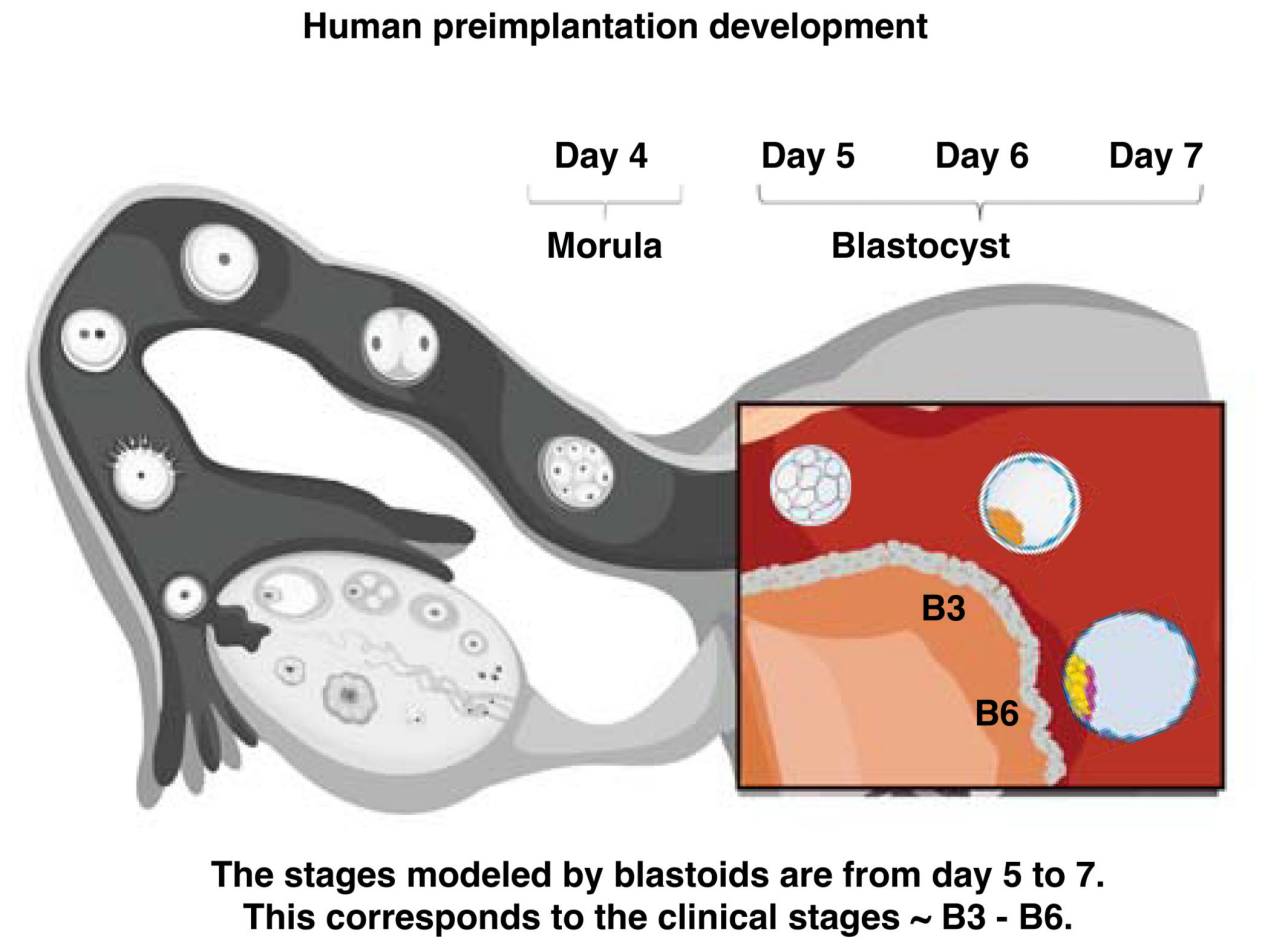
A schematic of the time window of human peri-implantation development modelled by blastoids. Five days after fertilization, the developing embryo is known as a blastocyst. Around this time, blastocyst ‘hatches’ into the uterine cavity and is ready to implant into the uterine wall. Implantation happens around 6-7 days after fertilization, but can only occur if the endometrial wall is prepared by the correct levels of hormones. Blastocyst usually implants with its inner cell mass facing the endometrium, known as the polar side. Human blastoids model the developmental window of days 5–7. This assessment is based on benchmarked morphological and transcriptomic data. The morphological evolution of blastoids reflect the clinical stages B3 to B6. Although blastoids might be co-opting some biological processes (*e.g*., Hippo inhibition for cell specification) occurring at earlier stages (*e.g*., morula to blastocyst transition), the projection of transcriptomic signatures of blastoid cells (24, 65, 96 hours) onto large reference maps including multiple stages of human embryonic development currently supports that blastoid cells resemble the cells of day 5 to day 7 embryos.

Human blastocyst development follows a particular pace and sequence. A human morula (day 4 post-fertilization) takes about 3.5 days to form a mature blastocyst (day 7)^23,24^ (Fig. 1). During that time, the embryo first generates the TE that forms an epithelial cyst with a fluid-filled cavity (blastocoel) and an inner cluster termed Inner Cell Mass (ICM)^24^ that attaches to one side of the TE. In a second step, the PrE and EPI forms from the ICM, and the polar and mural regions of the TE mature to define the embryonic-abembryonic axis^23,24^. This axis patterns the cellular functions necessary for implantation^26^. Modeling blastocyst development requires the timely recapitulation of this sequence of cellular commitment, morphogenesis, and patterning events^27^. By matching the pace and sequence of blastocyst development, cells similar to those of the blastocyst form (Fig. 1). Forming bona fide blastoids is crucial as a lack of fidelity in the developmental tempo, lineage commitment processes (specification and determination), final cellular proportions and states composition will reduce or abrogate the model’s predictive power to reveal mechanisms of development and diseases.

In the first part of this protocol, we will describe in details the initial parameters enabling human blastoid formation and then propose assessment criteria and methods for their analysis (Box 1). We propose 3 initial experimental parameters that, when appropriately tuned and combined, are sufficient to form blastoids that model blastocyst-stage embryos: (i) the initial state of the hPSCs (either human embryonic stem cells (hESCs) or induced PSCs (hiPSCs)), (ii) the initial size of the free-floating cellular aggregate, and (iii) the initial molecular environment the aggregate is exposed to. These 3 parameters respectively ensure an adequate intrinsic cellular potential, the spontaneous establishment of positional information, and a signaling environment (intrinsically created & extrinsically imposed) sufficient to trigger the cascading sequence of cellular commitment, morphogenesis, and patterning generating the blastocyst.

#### First parameter - The initial hPSC state

Depending on the culture conditions, hPSCs can be captured in states reflecting different and relatively discrete developmental stages^28^. To model the entire window of blastocyst development, this initial state should ideally reflect a stage preceding the blastocyst. At the moment, capturing stable cultures of stem cells homogeneously reflecting the pre-blastocyst stage remains an on-going endeavour^29–31^. However, hPSCs have been constrained into a ‘naïve’ state that transcriptionally reflects the blastocyst-stage EPI, for example when cultured in PXGL (PD0325901, XAV-939, Gö 6983, LIF)^32,33^. Other evaluation criteria including DNA methylation, X chromosome state, and transposon expression confirmed this resemblance^34–36^. Interestingly, the early human blastocyst contains cells that are still labile, undetermined, and that maintain a capacity to form TE^20,23,37^. Mirroring this plasticity, PXGL hPSCs are specified but undetermined as they maintain the capacity to form TE analogs^19,20,38^, a process that is guarded by the PRC2 complex that methylates histones to silence the expression of TE genes^39,40^. They can thus be used to form blastoids, with some limitations in modeling the early blastocyst stage (see below). On the contrary when hPSCs are cultured using either Fibroblast Growth Factor (FGF) 2 and Activin^28^ (‘primed state’) or LIF, CHIR99021, (S)-(+)-dimethindene maleate and minocycline hydrochloride^41^ (‘extended pluripotent state’)^41^, they reflect the post-implantation stage (see transcriptome analysis in^42–45^). In our original publication and in this protocol, we used PXGL hESCs and hiPSCs because they rather homogeneously reflect aspects of the undetermined blastocyst EPI, and have been successfully derived directly from blastocysts^20,46,47^. Other culture methods that capture ‘naive’ hPSCs might be suitable as well (*e.g*.,^36,48^), although blastoid efficiency, cell state, and genomic integrity may be different. Of note, all ‘naive’ culture conditions induce some level of chromosomic instability and thus remain sub-optimal. We have also discussed here^49^ the importance of the initial cell state for human blastoids.

#### *Second parameter* - The initial cellular aggregate size

During the morula to blastocyst transition, the cells located on the outer part of the aggregate have a free-edge membrane. This allows for the self-assembly of an apical domain including the atypical protein kinase C (aPKC). This is sufficient to inhibit the Hippo pathway thus freeing YAP1 to enter the nucleus and partner with TEAD4 to activate the transcription of the TE transcription factor CDX2 and GATA3^2,50,51^. The process of apical domain self-assembly is regulative by nature and depends on the plane of cellular division and on the subsequent inner or outer location of daughter cells^52^. Thus, the number of TE cells that form depends on the surface-to-volume ratios imposed by the size of the aggregate. These mechanisms have been mostly elucidated using mouse embryos, but they appear to be partly conserved in cows and humans^2,53–55^. Accordingly, we have shown that, during human blastoid formation, (i) aPKC and F-actin expression domains are co-aligned in the outer cells that also accumulate the Hippo downstream effector YAP1 and the TE transcription factors GATA2 and GATA3 in nuclei, in contrast with the expression of the EPI transcription factor NANOG. (ii) A specific aPKC inhibitor (CRT0103390) prevents YAP1 nuclear accumulation, decreases the number of GATA3+ cells and impairs blastoid formation. (iii) On the contrary, ligands of lysophosphatidic acid (LPA) receptors (LPA and NAEPA) that can inhibit the Hippo pathway^26,56^ enhance blastoid formation. (iv) Finally, the overexpression of wild-type or constitutively active forms of YAP1 (5SA) accelerates blastoid cavitation, while over-expression of YAP1 with a mutation in the TEAD binding site (S94A) doesn’t. We concluded that, during blastoid formation, the aggregate size imposes positional information (*e.g*., inner-outer) that balances the number of TE and ICM analogs through a co-optation of the Hippo pathway^2,53–55^. In our original protocol, we controlled the aggregate size using 96 well-plates hydrogel microwells made in-house^57,58^. In this protocol, we also used commercial Elplasia® 96 well-plates from Corning®, Gri3D® 96-well plates from Sun Bioscience SA, and AggreWell™ 24 well-plates from Stemcell Technologies (see comments in Supplementary Table 1).

#### Third parameter - The molecular environment the aggregate is exposed to

Beyond the positional information imposed by the aggregate, it remains necessary to initially stimulate hPSCs with external molecules. Because PXGL hPSCs reflect an EPI stage whose fate is specified but not determined^38^, cells must erase their specification choice to form TE and ICM analogs. This can be done using inhibitors of signaling activity. The inhibition of the Hippo pathway can be triggered using GPCR ligands^56^, and LPA had proved useful in regulating CDX2 and capturing mouse trophectoderm stem cells (TESCs) reflecting the blastocyst-stage^26,59^. In addition, it was known that ERK and TGFb inhibition direct hPSCs to specify a trophoblast fate^16^. When the initial hPSCs reflects the post-implantation EPI (e.g., formative and primed hPSCs), amnion and trophoblast analogs are generated that also reflect a post-implantation stage^16,19,20,60,19.^ However, when starting with t2iLGo or PXGL hPSCs that reflect the pre-implantation EPI, blastocyst-stage TE analogs are specified^19,20^. On these basis, in our initial publication, we used LPA, PD0325902 and A83-01 to triply inhibit the Hippo, ERK, and TGFb pathways. This triple inhibition is subtle enough that it does not override positional information and does not trigger TE specification of all cells within the aggregate, thus enabling the three lineages to form. More stringent inhibitions of the Hippo (using XMU-MP-1) and STAT pathways (using SC144) result in the formation of trophospheres mostly devoid of EPI analogs^61^. We also used the ROCK inhibitor Y27632 to improve aggregation^62^ and the STAT pathway activator Leukemia inhibitory factor (LIF) that is used to maintain a blastocyst stage EPI-like state^33^ and also improved the efficiency of blastoid formation. Altogether, the medium contains 5 molecules (PD0325902, A83-01, LPA, LIF, Y27632) and is termed PALLY. Note that only one medium is used that is sufficient to initiate the cascade of cell commitment, morphogenesis, and patterning.

Of note, testing scientific and biomedical hypotheses, for example using drug and genetic screens, requires the use of minimal culture media deprived of elements potentially interacting or masking the effect of an investigated molecule or genetic modification. Therefore, it is important to keep to a minimum the number of molecules and the duration of their presence during blastoid formation. Following our original publication, we now show that stimulation during the first 48 hours followed by the use of minimal stem cell medium (N2B27 minus insulin medium) or of clinical IVF media (G2 from Vitrolife, Continuous Single Culture-NX from Fujifilm, ORIGIO® Sequential Blast™ from CooperSurgical Fertility) also supports efficient blastoid formation. Also, we show that human blastoids can be cryopreserved and thawed using standard IVF procedures, while retaining very high viability, thus allowing for shipping and for optimizing blastocyst vitrification/thawing.

These initial experimental criteria are sufficient to efficiently trigger hPSCs to re-enact the first step of TE/ICM commitment. Beyond this, the second landmark of blastocyst development is the specification of the EPI/PrE and patterning of the TE into the polar and mural regions based on the distance to the EPI. Altogether, this second step defines the first axis, termed embryonic-abembryonic, which allows for the acquisition of the different TE functions necessary for implantation in utero. Axis formation includes a process by which the human EPI induces the local maturation of the polar TE (pTE) that subsequently acquires the capacity to attach to the endometrium, the uterus lining^61^. In the mouse, it also includes a process by which the EPI maintains CDX2 expression in the pTE to produces Wnt6/7b that instruct the uterine cells to form a decidua (uterine cocoon)^26^. Using this protocol and based on the markers CDX2 and NR2F2, we measured that >60% of blastoids also go on to spontaneously form tissues similar to the polar (NR2F2+/CDX2-) and mural TE (NR2F2-/CDX2+)^23,61^.

Altogether, this protocol allows for the formation of blastoids that follow the pace and sequence of blastocyst development. As a consequence, we and others have measured by scRNAseq that the cells formed reflect the blastocyst-stage embryo^61,63,64^ (see analysis method below and here^49^).

Contrary to mouse blastoids, it is ethically unacceptable and forbidden by the ISSCR to transfer human blastoids inside any uterus^65,66^. As an alternative, we tested the functions of human blastoids by developing an *in vitro* implantation assay using primary endometrial organoids. Previously, cell lines from endometrial adenocarcinoma (Ishikawa^67,68^, ECC-1, RL95-2, or HEC-1-A^69^) have been combined with human blastocysts. However, due to their malignant origin, these cells have a limited potential to recapitulate the specificity of implantation (*e.g*., species specificity^70^, hormone priming specificity^71^). As an improvement, endometrial organoids enable the long-term growth of small biopsies in 3D Matrigel culture, contain diverse primary cell types, and retain the biological properties and genetic stability of the original tissue^72,73^. We used these cells in a 2D assay, which we called open-faced endometrial layer (OFEL) and opens up the apical surface of epithelial organoids facilitating blastoid deposition and analysis (*e.g*., live imaging). *In utero*, during the menstrual cycle, the endometrium is made receptive by a combination of estrogen (E2), progesterone (P4), the downregulation of the WNT signaling pathway and the enhancement of cAMP signaling, both induced by the decidualized stromal cells^74^. This defines a moment known as the window of implantation (WOI) permits blastocyst implantation^75–77^. On the basis of these previous findings, we primed OFELs with E2, P4, a WNT inhibitor (XAV939), and cAMP, which accordingly increased markers of the WOI^61^. Interestingly, human blastoids deposited on such endometrial layers attached only when these layers were hormonally primed, and the contact specifically occurred *via* the polar side of the blastoids, as *in utero*. Upon attachment, the pTE repelled the endometrial cells, a process that is necessary for the blastocyst to invade the deeper uterine layers. We further assessed the specificity of these interactions between the pTE and the primed endometrial cells by testing the role of EPI inductions. Adding a GP130 inhibitor (SC144) or a potent inhibitor of the Hippo kinases MST1/2 (XMU-MP-1) during blastoid formation resulted in the formation of trophospheres mostly devoid of EPI analogs. Although these trophospheres transcriptionally reflected early and late blastocyst TE, they failed to attach to endometrial cells. We therefore concluded that signals from the EPI induce the local maturation of the pTE, which endows them with the capability of attaching to the uterus lining. After attachment and upon extended culture, blastoids expanded and formed multiple relevant cell types including trophoblasts expressing hCG at levels detectable using standard pregnancy tests. Overall, beyond some minimal blastoid criteria (morphometry, efficiency, timing, sequence of lineage commitment, axis formation, see Supplementary Table 2^61^ and ^27,49^), the observation that, like blastocysts, blastoids attach only to hormonally primed endometrial cells, and specifically *via* the pTE analogs increases the confidence in the functionality of the model, and opens possibilities to mechanistically investigate early human implantation and development.

Since our original publication, we have improved the method for OFEL culture. In this updated protocol, we start the priming of the cells later, only during the 2D OFEL culture. In brief, this process involves enzymatic and mechanical dissociation of organoids into single cells or small colonies which are plated onto Matrigel-coated plates for further expansion. Once confluency is reached, E2 supplemented medium is added for 2 days to support cell proliferation and to prime the cells for progesterone stimulation. Thereafter, OFELs are stimulated with E2, P4, cAMP, and XAV-939 for 4 days to make OFELs ready for an implantation assay. Here, we provide detailed methods for culturing endometrial organoids and OFELs, and depositing blastoids for an implantation assay.

### Overview of the procedure

**Fig. 2:**
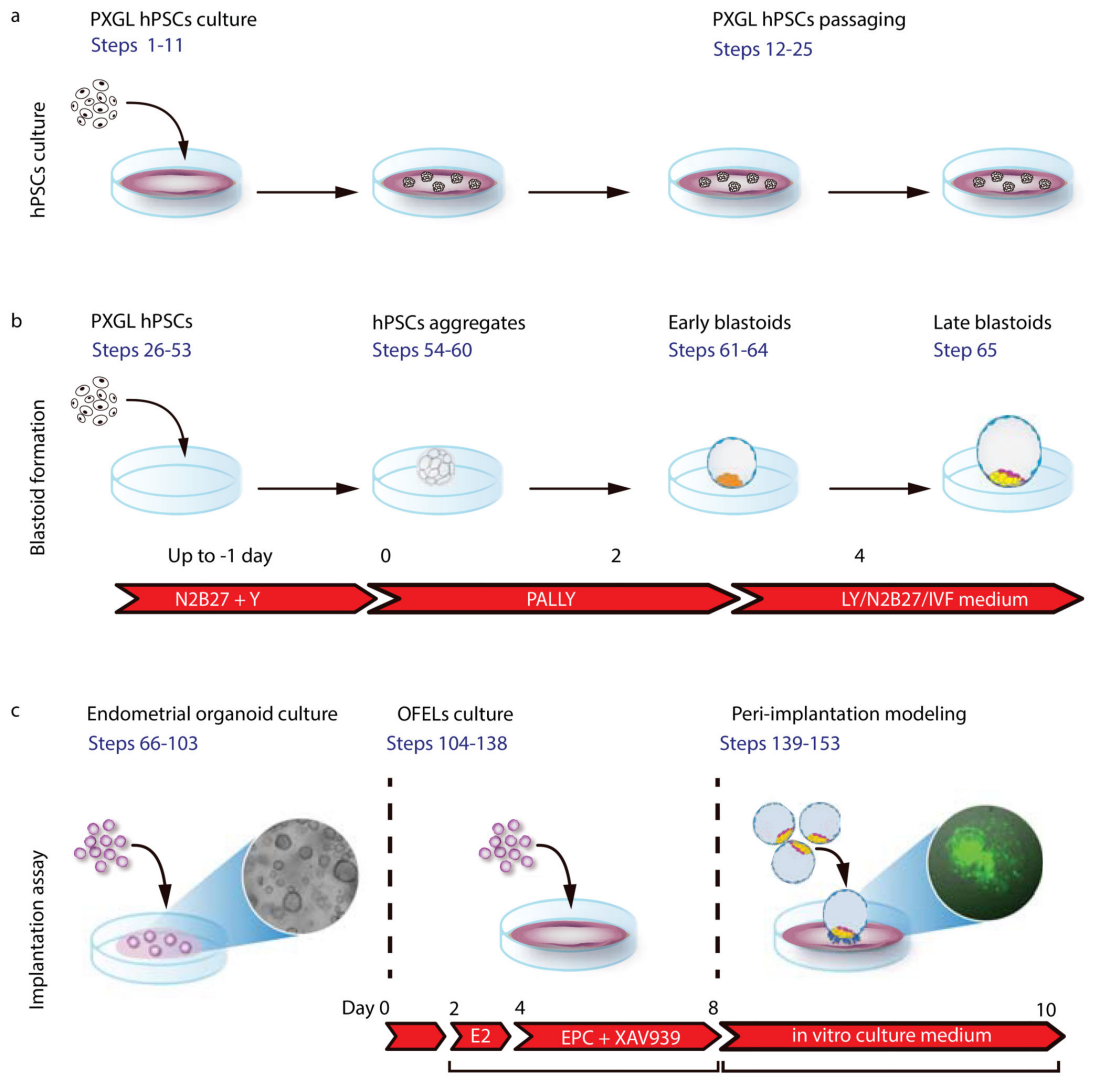
Schematic overview of the protocol. The workflow includes; a, initial PXGL hPSCs seeding and passaging (Steps 1-25), b, procedure for the formation of human blastoid from hPSCs cultured in PXGL medium (Steps 25-65) and c, Seeding and passaging endometrial organoids (Steps 66-103), OFEL formation (Steps 104-125) and Implantation assay (Steps 126-138) a, Human embryonic stem cells (ESCs) or induced PSCs (iPSCs) cultured in PXGL medium are seeded into feeder-layers for maintenance and passaged several times before human blastoid formation (Steps 1-25). b, hPSCs are dissociated into single cells (Steps 35-47) and plated in non-adherent hydrogel microwells or AggreWell plate to form small aggregates of the cells (steps 48-53). Upon exposure to lysophosphatidic acid (LPA), A83-01 and PD0325901 in a chemically defined medium containing leukaemia inhibitory factor (LIF) and Y-27632 for 2 days, some aggregates form small cavities (steps 54-61). With continuing culture for 2 days in a medium containing LPA and Y-27632, human blastoidss are formed efficiently and consistently (Steps 62-65). N2B27, serum-free medium; PALLY, PD0325901 + A83-01 + LPA + hLIF + Y-27632; LY, LPA + Y-27632. c, Procedure for the formation of open faced endometrial layer (OFEL) from human endometrial organoids. Human endometrial organoids are seeded and passaged before OFEL formation (Steps 66-103) and then are dissociated into single cells and small colonies and plated in Matrigel-coated wells (Steps 104-120). After 1-2 days, the cells become confluent and grow as a monolayer. Once confluency is reached, E2 supplemented medium is added to the cell for 2 days (Steps 121-122) and then, OFELs are stimulated with E2, P4, cAMP, and XAV-939 for 4 days to make them ready for implantation assay (Steps 123-125). In the next step, human blastoids that display blastocyst morphology with compact ICM are selected and transferred onto OFELs (Steps 126-135). Attachment and development of blastoids will be monitored in the next days (Step 136). OFEL, open face endometrial layer. EPC, E2+P4+cAMP.

In this protocol, we provide step-by-step guidance to form human blastoids that recapitulate several key features of human blastocyst development and implantation, as described above. The workflow of the protocol is depicted in Fig. 2. It is separated into 5 different stages. (i) The first stage covers the 2D culturing of the hPSCs (steps 1-25). In this part, we provide the detailed protocol for thawing, passaging and maintaining the cells in the optimal state on feeder-layers, as well as detailed instructions for hPSCs cryopreservation (Box 2). (ii) The second stage explains the generation of human blastoids (steps 26-65). In this part, we describe in detail how to generate human blastoids using multiple commercially available plates and multiple cell lines, and provide troubleshooting tips. We also provide details for switching to minimal stem cell or clinical IVF media (step 63), and for blastoids cryopreservation (Box 3). (iii) The third stage describes the culturing of human endometrial organoids (steps 66-103). We provide detailed instructions for the thawing of frozen vials, passaging, expanding, and cryopreservation of the endometrial organoids (Box 4). (iv) The fourth step explains the generation and priming of OFELs for the blastoid implantation assay (steps 104-125). We describe how to generate OFELs using endometrial epithelial cells previously maintained as organoids and provide details for cell seeding and hormonal stimulation to prepare the primed OFEL layers (v) Finally, the fifth step describes the implantation assay which combines blastoids and primed OFELs (steps 126-138). In this part, we provide additional instructions on how to obtain a confluent OFELs ready for the implantation assay, and the procedure to select blastoids and transfer them onto the OFELs. We also detail the procedure for immunofluorescence staining of blastoids (steps 139-153). All steps must be followed precisely to ensure a successful outcome. It requires practice and attention to details. Based on our experience, we anticipate that a person with about 1 year of hPSCs culture experience should be able to perform the protocol, including blastoid formation, OFEL preparation, and subsequent analysis.

### Target audience and applications

Our protocol provides a platform for scientists in both basic (*e.g*., developmental biology) and biomedical sciences (*e.g*., preclinical drug testing and disease modeling). Knowledge of the mechanisms acting at the onset of embryonic development offers opportunities to develop therapeutics (i) improving public health through effective family planning, (ii) reducing an ongoing global fertility decrease with profound economic, social, environmental, and geopolitical consequences^78^, and (iii) limiting the appearance of chronic diseases later in life through prenatal preventive medicine (see the developmental origin of health and diseases)^10,11^. Because blastoids can be generated from established cell lines and don’t necessitate the repeated use of embryos, they represent an ethical opportunity complementing research using donated IVF embryos^65^. Moreover, since there are major differences in pre-implantation development and implantation across mammalian species, the generation of blastoids from other mammals will be useful for audiences who interrogate the evolution of developmental mechanisms, forms and functions.

### Current limitations of the protocol and potential future directions

We identify three main limitations that have also been discussed previously^79^. First, the initial number reflect the early-to-mid blastocyst stage (E5-6) and the transcriptome of the PXGL hPSCs and subsequently the early blastoid cells (within 24 hours after triple inhibition) reflect the EPI same stage. Both the initial cell number and transcriptome does not reflect the morula stage (10-16 cells, E4). As such, PXGL hPSCs, whose fate is specified but not determined, mimic some aspects of TE fate specification, but don’t recapitulate the overall morula-to-blastocyst transition. In mice, this transition is defined by the compaction of the morula (8+ cells) that leads to the concomitant inhibition of the Hippo pathway, polarization/epithelization, TE specification of the outer cells, and formation of the blastocoel cavity^80^. Human morulae compact relatively later (10+ cells) and, although cells transcriptionally progress between the late morula stage and early human blastocyst stage, early human blastocyst cells are not committed yet. This was carefully shown (i) by combining scRNAseq pseudotime with video annotations^23^, (ii) by showing that cells dissociated from early blastocyst have the potential to form blastocysts *de novo*^37^, and (iii) by showing that immunosurgically isolated ICM/EPI from late blastocysts (>E5-6) remain capable of forming GATA3+ TE analogs^20^. In other words, human cells form a blastocoel cavity before the TE is specified, and ICM/EPI from late blastocyst are undetermined as they remain capable of forming TE cells. The delayed TE/ICM fate specification relatively to embryo morphology and delayed determination of the EPI in humans as compared to mice is mirrored *in vitro* by the capacity of PXGL hPSCs to generate TE analogs^19,20^. On the contrary, mouse PSCs have determined their fate and lost the capacity to form trophoblasts^31^. We have shown that hPSCs co-opt and leverage the inhibition of the Hippo pathway to specify the TE analog, as during morula-to-blastocyst transition^61^ and that the Polycomb Repressive Complex 2 is involved in TE commitment^39,40^. However, although blastoids co-opt some TE specification mechanisms (e.g., Hippo inhibition), they don’t recapitulate the global transcriptome shift occurring between morula-to-early blastocyst stages, and mostly recapitulate aspects of the early to late human blastocyst development (E5-7).

Second, the reference map of human embryos (E3-16), which is essential to evaluate embryo models, remains incomplete and fragmented. In order to more finely assess the stages and lineages captured by embryo models, it will be necessary to increase the granularity of this reference map by adding cells harvested at more precisely defined stages (*e.g*., by combining time-lapse imaging of annotated embryos and transcriptomic analysis^23^). Especially, the addition of more amnion cells is necessary to better distinguish them from trophoblasts, as both cell types are transcriptionally close^60^. There is also a necessity to obtain additional layers of stage-specific information (*e.g*., total RNA^81^, non-coding RNA, epigenetic state^82^, X chromosome state, proteomic state). Such finer and more complete reference maps and analysis methods (e.g., adequate parametrization of the merging of datasets as explain previously^64^) will be key to further establish the similarities and differences between blastocysts and blastoids. These similarity levels will assert the predictive value of the model, guide the scientific questions that can currently be answered, and inform on the ethical status of embryo models^79^.

A third limitation lies in the implantation assay which is of 2D nature and does not include uterine cell types other than the endometrial epithelial cells. Upon interaction with the endometrium, the blastocyst attaches to and repulses the uterus lining to invade the underlying layers that include stromal, endothelial, and immune cells. The stromal and glandular cells are especially important as they undergo a process called decidualization to nurture (*e.g*., secreted growth factors) the implanting blastocyst. The current implantation assay allows modeling the initial step of attachment and repulsion, but not the invasive processes and interactions with underlying uterine cells. The 2D nature of this assay also strongly limits the correct development of blastoids and blastocysts through the post-implantation stages. Future research to produce a physiological model of the uterus will involve developing 3D chemically-defined matrices recapitulating the luminal and glandular architectures of the endometrium, and incorporating underlying stromal cells, perfused endothelial cells, immune cells, and microbiome that all contribute to the implantation process. Engineering will be key to form a niche that mimics aspects of the dynamic and complex uterine environment.

### Comparison with other methods

**Fig. 3:**
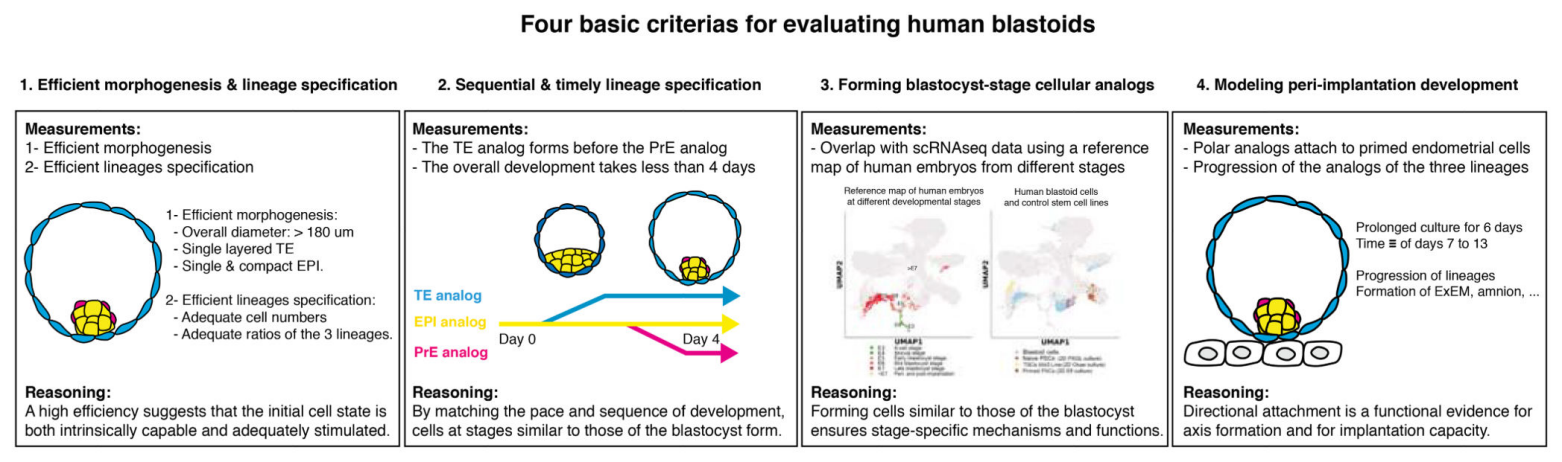
Four basic criteria to validate the formation of blastoids. Human blastoids should comply to 4 basic criteria (see also Comparison with other methods in the text). (i) Blastoids should form efficiently both in terms of morphology and of specification of the analogs of the 3 lineages, EPI (yellow), PrE (magenta), TE (cyan). A high efficiency suggests that the initial cell state is both intrinsically capable and adequately stimulated. (ii) Blastoids should generate analogs of the 3 lineages according to the developmental sequence (TE/EPI first, pTE/PrE second) and pace (< 4 days) of blastocyst development. By matching the pace and sequence of development, lineages and cells similar to those of the blastocyst form. (iii) Blastoids should form blastocyst stage cellular analogues, but not of post-implantation stages as defined by transcriptome comparison through scRNAseq. To do so, a reference map of human embryos at different stages is essential as using a map restricted to the targeted cells prevents revealing the presence of off-target cells. A reference map is available here: https://petropoulos-lanner-labs.clintec.ki.se/app/shinyblastoids and is used in this figure. Forming cells similar to those of the blastocyst ensures stage-specific mechanisms and functions. (iv) Blastoids should be capable of recapitulating functional features of blastocyst implantation and development. EPI (yellow), PrE (magenta), TE (cyan). Directional attachment of blastoids specifically to primed endometrial layers is a functional evidence for axis formation and for uterus implantation capacity.

In 2021, attempts to form human blastoids were made^21,61,83–86^ that are currently being evaluated for the developmental stages and lineages they reflect^63,64,87^. These attempts pinpointed the initial parameters necessary to form blastoids (see above) and also allowed for the establishment of minimal criteria to assess the creation of a blastoid. These criteria include the morphology (*e.g*., the mean diameter of a mature blastocyst is 193+/-18 um^88^), the number of cells expressing lineage-specific transcription factors (e.g., CDX2 for TE, Nanog for EPI, Sox17 for PrE), the sequence of lineages specification (TE/EPI first, pTE/PrE second^23^), the time of formation of a morphologically correct structure (∼3.5 days^23^), and the level of overlap of the transcriptome of the three lineages with the blastocyst^64^. Such initial parameters and assessment criteria have been summarized here^61^ (Supplementary Table 2 therein) and here^49^, commented here^27,79,89^, and are also summarized in Figure 3 and Box 1.

In light of these criteria, a first simple way to evaluate the quality of a protocol or experiment is to look at the **efficiency of formation of blastoids** (based on the precise morphological criterias of blastocysts). A high efficiency suggests that the initial cell state is both intrinsically capable and adequately stimulated. Only a few carefully curated molecules are sufficient. Using extra molecules might slow down the morphogenetic processes and lead to the formation of abnormal or differentiated cell types reflecting later embryonic stages. The use of each molecule should be compelling as unjustified ones might affect the predictivity of the assay or mask the effect of molecules or gene products whose function is being tested. A second simple way to evaluate a protocol or an experiment is to look at the **time necessary for the blastoids to form**. A human morula takes about 3.5 days to form a late-stage blastocyst^23^. If morphogenesis takes longer, then hPSCs are likely to generate abnormal or more differentiated cells reflecting later embryonic stages. In other words, the timing of morphogenesis must be coupled with the commitment of the lineages to match developmental pace. The third way to evaluate an experiment is to measure the **similarity of the transcriptome of blastoid and blastocyst cells**. This method is more expensive/time consuming, as well as more difficult to analyze but is facilitated by the use of a reliable reference map of the early human embryo. To facilitate benchmarking, an international consortium of scientists has built a reference map that can be used to project and evaluate the stage of the generated cells, and to infer the level of analogy to blastocyst cells^64^. Although this reference map must be improved, it provides a state-of-the-art merging of cells harvested from both human embryos and models at different stages. Importantly, using a reference map covering a wide range of stages (e.g., pre- and post-implantation) is essential for proper benchmarking^64^, while a reference map covering only the targeted stages (e.g., blastocyst cells) prevents the identification of off-target cells. We and others^61,63,64^ have measured that blastoids generated using this protocol lead to the formation of >97% of cells whose transcriptome reflect the blastocyst stage with the majority of the <3% of off-target cells being similar to the extraembryonic mesoderm^63^. A next validation step consists in combining single cell RNA sequencing analysis with multiplexed RNA/protein stainings for the visualization lineages- and stage-specific markers (*e.g*., LAMA4^24^/Nodal for the blastocyst-stage ICM/EPI, CDX2 for the blastocyst-stage TE, see https://petropoulos-lanner-labs.clintec.ki.se/app/shinyblastoids). Of note, the expression of these markers is not sufficient to assess a stage and transcriptome wide similarity remains the method of reference. Also, genes are used multiple times during development and are not exclusive to a tissue and stage. For example, CDX2 is also expressed both in the early TE and in the amnion, but not in the post-implantation trophoblasts.

When the initial 3 parameters are inadequate, although a fluid-filled cavity might form, the cells’ transcriptome doesn’t reflect the blastocyst stage but rather post-implantation developmental stages, including gastrulation (E14), germ layers (mesoderm and endoderm), amnion, and post-implantation trophoblasts^64^. ‘Looks can be deceiving’ as many other epithelial tissues (*e.g*., PrE or post-implantation EPI) can form cysts, and the formation of a cavity is not sufficient to allege blastoid formation. A lack of fidelity in the developmental processes and cellular composition will reduce or abrogate the model’s predictive power to study underlying mechanisms. Altogether, the 2021 attempts contributed in establishing necessary standards for human blastoids^79,90^.

## Experimental design

### Ethical approval

#### License for human blastoid research

Before starting research using human blastoids, it is essential to obtain ethical approvals from the relevant national and/or local/institutional authorities. According to the ISSCR guidelines 2021, human blastoid research should be overseen by the most relevant ethical committee, when possible the same one that oversees research done on human embryos^65,66,91^. The *Austrian Academy of Sciences* (local ethical body) has given our laboratory a license to perform these experiments, following expert legal advice that concluded these are not in conflict with Austrian laws. This license is in the shape of a statement of the Commission for Science Ethics of the Austrian Academy of Sciences concerning the project “Modeling human early develoment using stem cells.” There is no approval number on that document. This license conforms with the ethical standards suggested by the ISSCR.

#### Right to use and registration of the cell lines

Blastoids. Wicell provided the H9 line of hPSCs, and was informed of the experiments performed *via* a ‘simple letter of agreement’ under the entitled program ‘*Modeling early human development; Establish a stem cell based 3D in vitro model of human blastocyst (blastoids)*.’ Wicell didn’t consider that, given the current developmental potential of blastoids, this research is contrary to the donor’s informed consent and therefore allowed its material to be used for current blastoid research. Wicell recognizes, however, that this may change in the future as research continues and the developmental potential of blastoids becomes more embryo-like. It provided notice that stem cell-derived embryo models, including blastoids, may progress to meet the definition of whole embryo in some of the informed consents (that is, would develop into a fetus if returned to the uterus). If used to make a whole embryo, Wicell specifically reserved the right to demand the immediate return or destruction of any such materials or modifications to materials and the retraction of any related articles. Wicell has not provided information about the criteria defining a tipping point when blastoids could eventually become considered as embryos. This research was funded by the European Research Council, and therefore all cell lines were registered in hPSCreg (https://hpscreg.eu/) under the project *BLASTOID, A discovery platform for early human embryogenesis*. Finally, the ISSCR guidelines also stipulates that it is forbidden to transfer human blastoids into any uterus or uterus explants either animal or human.

Endometrial organoids. Ethical approval from the designated ethical committee at the Royan Institute in Tehran for deriving endometrial organoids and informed consent from patients were both sought when obtaining endometrial tissue (IR.ACECR.ROYAN.REC. 1397.93). In addition, acquisition of human endometrial samples conformed to relevant institutional and national regulations and to informed patient consent that were obtained to use the endometrial organoids.

#### Human blastoids and the 14-days rule

Although blastoids are not considered as embryos by the ISSCR guidelines and by the providers of the H9 cell line, and although the ISSCR guidelines don’t enforce a maximum time for the *in vitro* culture of human embryos, we did not pass the developmental time-equivalent of 14 days after fertilization, which is characterized by the initiation of the formation of the primitive streak. Blastoids are morphologically and transcriptionally similar to day 7 embryos, and were cultured for an additional 6 days thus reaching a culture time-equivalent of 13 days. Of note, the number of cells and their organization did not reflect the developmental stage of a day 13 human embryo. It is currently unclear when divergence from normal development occurs. However, the fact that blastoids are cultured in 2D, when they should invade a 3D uterine-like environment, largely prevents normal development.

### Culturing of hPSCs

**Fig. 4:**
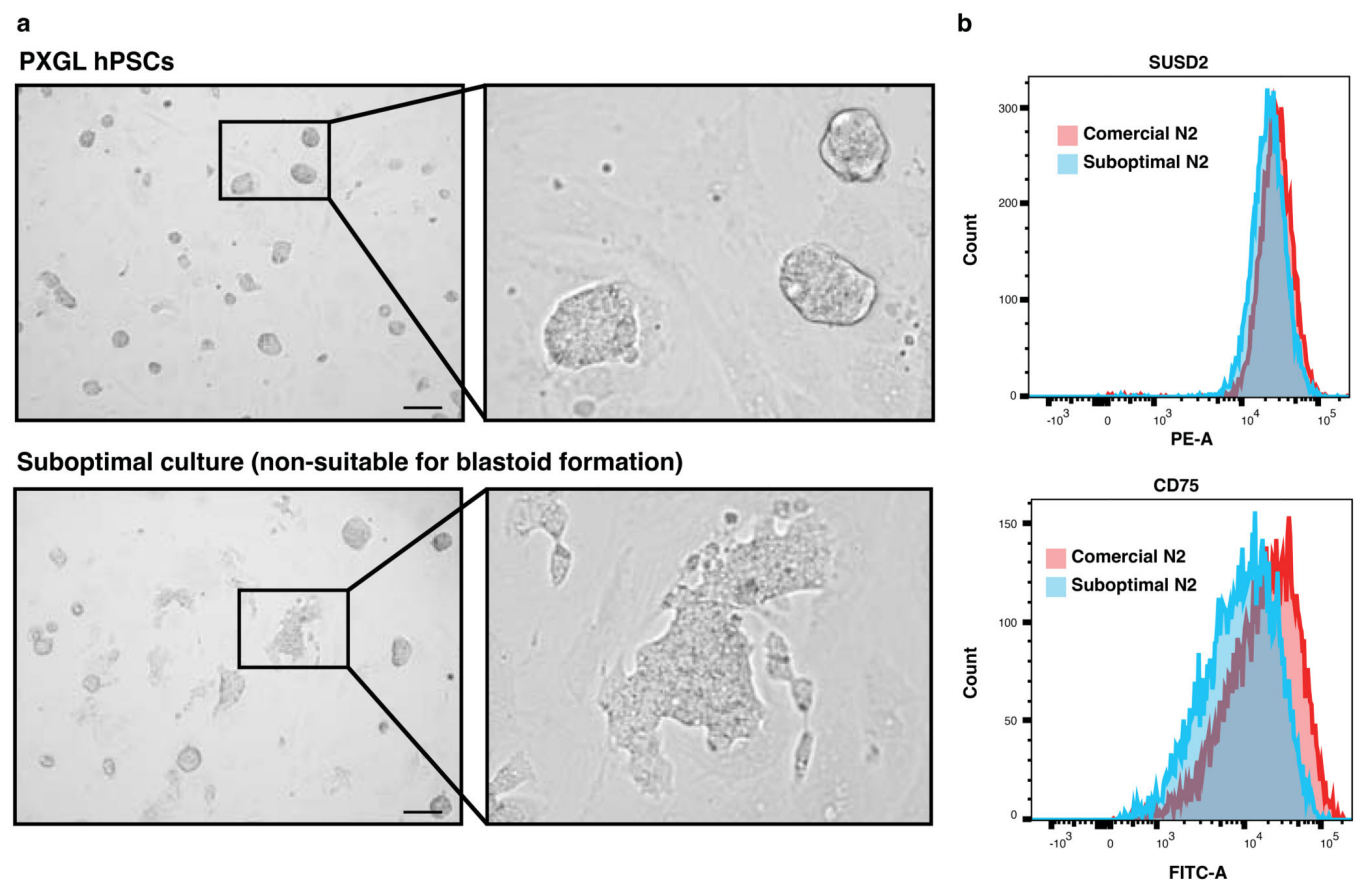
Morphological characterization of PXGL hPSCs. a, Representative images of PXGL hPSC colonies, cultured in an optimal hPSC medium (PXGL) and suboptimal culture condition. In the suboptimal culture condition, hPSCs will spontaneously differentiate as seen by loss of colony border integrity, loss of dome-shaped morphology and exhibition of a flat morphology. Scale bars, 100 μm. b, Flow cytometry analysis plot of hPSCs cultured in optimal and suboptimal culture conditions and stained with specific antibodies (SUSD2 and CD75).

Human blastoids can be established from either human embryonic stem cells (ESCs) or induced PSCs (iPSCs). There are several factors that the experimenters should be aware of when preparing to run the protocol, such as characteristics of individual cell lines, feeder cells, reagents and equipment. We use PXGL medium and feeder-layers for hPSC maintenance. It is crucial to maintain hPSCs in an undifferentiated state. hPSC colonies typically have a dome shaped morphology with bright and defined borders (Fig. 4a). hPSCs should be cultured with Y-27632 for 24 hours after every passage. If the quality of cell culture decreases based on the cell morphology (*e.g*., emergence of flat colonies in the population), adding Geltrex (0.5 µl/cm2) to the medium during the first 24 hours after passaging can increase the quality of cell culture. Geltrex is a reduced growth factor Basement Membrane Matrix including laminin, collagen IV, entactin, and heparin sulfate proteoglycans. It enhances the attachment and maintenance of hPSCs.

The density of hPSCs in culture is another crucial parameter for maintenance of the hPSC state. The cells grow very slowly at low density, especially during the first passages after thawing, and spontaneous differentiation increases as the density of the cells increases. The feeder cells also need to be at optimal density when hPSCs are seeded. If the density of the feeder cells is too low, the hPSCs will spontaneously differentiate as seen by loss of colony border integrity, loss of dome-shaped morphology and exhibition of a flat morphology (Fig. 4a). In case of too high feeder cell density, the hPSCs grow slower. We recommend using MEFs isolated from E13 CF-1 mouse embryos as a feeder layer. Due to inherent batch differences, we also recommend testing the ability of each batch of MEFs to support hPSCs growth and maintenance.

Markers of the human ICM and blastocyst-stage EPI are necessary to authenticate the quality of the cultures of hPSCs. Although these markers are currently limited, hPSCs can be regularly authenticated for their state using a SUSD2/CD75^64,92,93^ FACS assay (Fig. 4b). Sorting of SUSD2-high cells can be used to improve cultures or to select for the pristine hPSCs during resetting. Additional markers, for example LAMA4 for the day-5 ICM have been proposed and should be tested^24^. PXGL hPSCs have been reported to maintain a normal karyotype^47^. However, aneuploid cells will arise overtime and monitoring genetic stability is important for all stem cell cultures. It is highly recommended to test for chromosomal abnormalities^94^ and for genomic deletion/duplication (*e.g*., using microarray-based Comparative Genomic Hybridization (aCGH)) before starting blastoid experiments.

### Human blastoid formation

**Extended Data Fig. 1:**
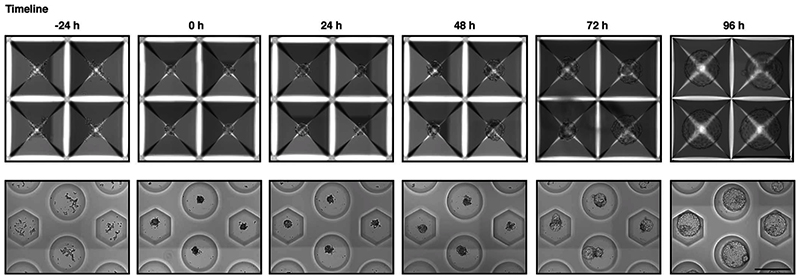
Triple inhibition of Hippo, ERK and TGFβ pathways leads to efficient and robust formation of human blastoids. Time course bright-field images of PXGL hPSCs aggregates and blastoid formation within AggreWell (top) and microwell arrays (bottom) in PALLY medium. Scale bars, 400 μm.

**Fig. 5:**
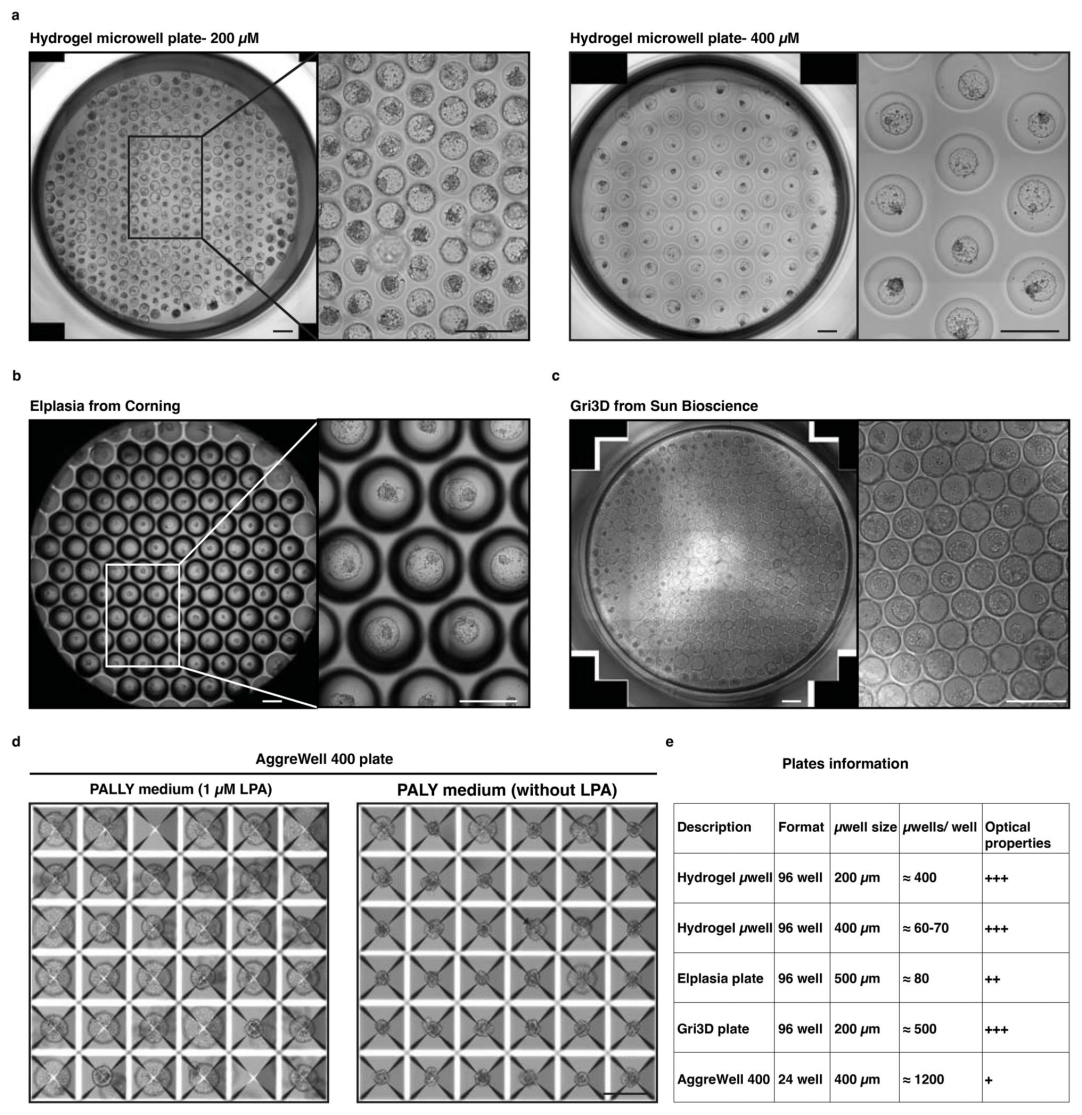
Triple inhibition of Hippo, ERK and TGFβ pathways leads to efficient and robust formation of human blastoids composed of blastocyst-like cells. Bright-field image of human blastoids formed innon-adherent hydrogel microwell plates (a), Elplasia plate (b) and Gri3D plate (c). Scale bars, 400 μm.d, Bright-field image of human blastoids formed in non-adherent AggreWell plates after 96 h either with LPA (PALLY medium, left) or without LPA (PALY medium, right). Each well of an AggreWell plate is 400 μm in diameter. Scale bar, 400 μm. e, Information for each plate used for blastoid formation. Hydrogel microwell plates and Gri3D plates have the best optical properties (+++).

**Fig. 6:**
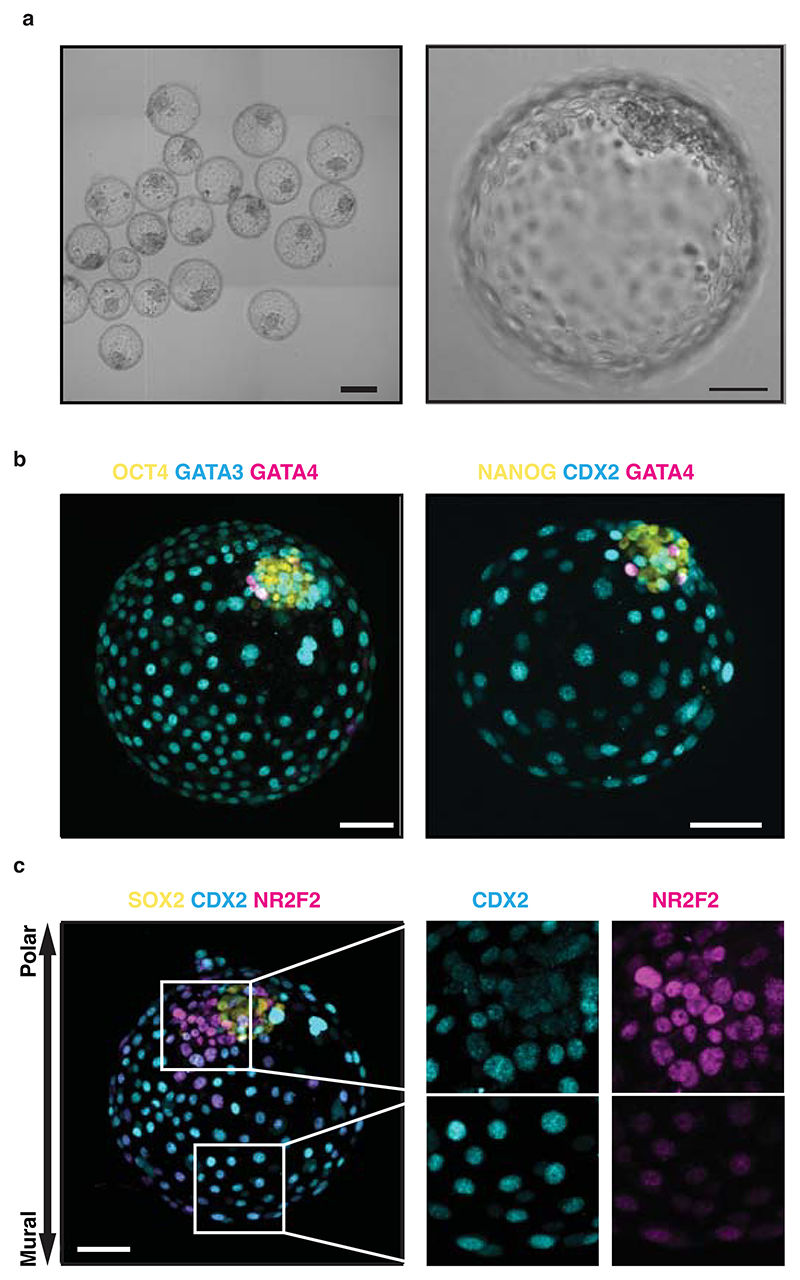
Human blastoids resemble the morphology of the blastocyst, comprise analogues of the three founding lineages and form an axis. a, Bright-field images of representative human blastoids harvested from wells (left) and representative image of a typical human blastoid (right), Scale bars, 200 μm (left), 50 μm (right) b, Confocal immunofluorescence image (MIP) of the epiblast (EPI) markers NANOG and OCT4 (yellow), the TE markers CDX2 and GATA3, and the PrE markers GATA4 (magenta) in human blastoids. c, Confocal immunofluorescence image (MIP)of CDX2 (cyan), Polar TE marker NR2F2 (magenta) and NANOG (yellow) in a representative human blastoid. Scale bars, 50 μm. MIP, maximum intensity projection.

It is crucial to start with a homogenous culture of high-quality hPSCs before blastoid formation. Therefore, all the MEFs and differentiated hPSCs need to be carefully separated from the pristine hPSCs. MEF feeders and differentiated cells can be excluded from the hPSCs culture by plating the cells onto gelatin coated plates as the majority of non-naive cells will be excluded by adhering. MEFs can be identified under the tissue culture microscope as larger cells as compared to hPSCs. After collecting high-quality hPSCs, it is critical to control cell number and aggregate size per microwells. Optimal initial cell number per microwell can vary among the different cell lines. For example, for the H9 and HNES1 cell line, 45 ± 10, and 55 ± 10 cells/microwell gives rise to high efficient blastoid formation respectively. As discussed above, the size of the aggregate is likely to impose an intrinsic positional information necessary for a selective Hippo inhibition and TE specification. An inappropriate starting cell number can result in a too small aggregate, which would prevent cavity formation, or in a too large aggregate, which would lead to the formation of cavitated structure with multiple cavities and overall blastoid diameter reaching more than 250 µm. In our lab, we control the initial cell number using hydrogel microwells made in house but here we show that other commercially available plates are perfectly suitable (see Fig. 5 and the ‘Reagents’ section). To successfully generate human blastoids, precise alterations of major signaling pathways (Hippo, TGF-β and ERK) is another critical step. The experimenters must be aware that outcomes are cell line dependent, and inconsistency is potentially due to the different genetic backgrounds and to differences occurring between different batches of resetting procedures. Therefore, some cell lines may require a slight modification to the optimal cell number and blastoid induction method. For example, even though treatment with PD0325901, A83-01, LIF and lysophosphatidic acid (LPA) from day 0 to day 2 is recommended as the standardized way in this protocol, optimization of concentrations and timing may be required to efficiently induce blastoid formation in other cell lines. The optimization process must be performed sequentially; for example, when optimizing timing of blastoid induction, the experimenters must keep concentrations and cell number unchanged. By comparing the morphologies under a microscope, it is possible to determine the optimal conditions in which the aggregates form blastoids with high efficiency. For example, for the H9 cell line, blastoid induction from the seeding time gives rise to highly efficient blastoid formation, while a short period of aggregation in minimal medium might be necessary for other lines. Blastoid formation is completed on Day 3-4 after induction (Fig. 5a-c and Extended Data Fig. 1) and it is routinely defined based on morphological similarities to B6 staged human blastocyst^88^, as a structure composed of a monolayered cyst with an overall diameter of 150–250 μm comprising one inner cell cluster,^61^ (Fig. 6). If the experiment is not optimal, the cell aggregates will fail to form cavities or form an epithelial cyst without an inner cell cluster.

### Endometrial organoid culture and OFELs formation

**Fig. 7:**
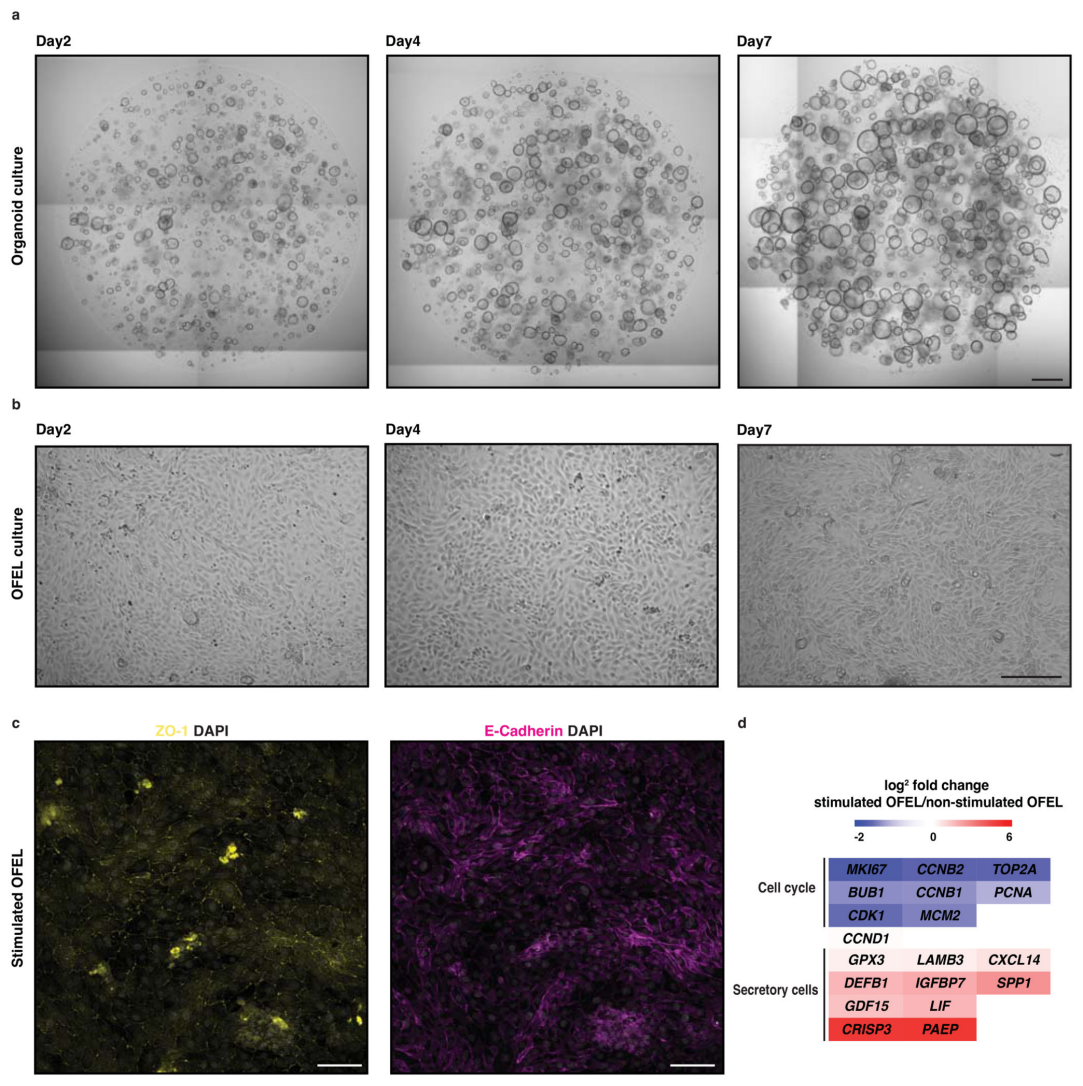
Representative images of endometrial organoids and monolayers. a, Bright-field images of time course organoid growth at days 2, 4 and 7 in Matrigel. Scale bars, 500 μm. b, Representative images of OFELs after 2, 4 and 7 days post seeding. Scale bars, 200 μm. c, Confocal immunofluorescence image for the tight junction molecule ZO-1 (Yellow), the adherence junction molecule E-Cadherin (Magenta) in representative stimulated OFELs. Scale bars, 100 μm. d, Heatmap of key cell cycle and secretory epithelial genes differentially expressed between stimulated and non-stimulated OFELs in bulk transcriptome. The endometrial cells were stimulated with hormones in the organoid culture and then were seeded to form OFELs.

We use already established endometrial organoids for OFELs formation. The detailed protocol for generation of endometrial organoids was previously published^72,95^. We recommend cryopreserving cryovials for each newly established organoid at early passages. The split ratio and passage interval of organoid differ among donors and should be optimized before OFELs generation. Endometrial organoids can be passaged every 7–10 days with a split ratio at 1:3–1:6. The best time to passage endometrial organoids and use them for OFEL is when organoids reach a diameter around 200 μm (Fig. 7a). We recommend moving to subsequent stages of the procedure when organoids reach the indicated size (typically around 200 µm) rather than at a particular time after the last passage. The best way to passage endometrial organoids is through dissociation into small fragments by mechanical and enzymatic disruption. We use Matrigel for endometrial organoid culture, however, Basement Membrane Extract (BME) can be used as an alternative. We use homemade Noggin and R-spondin-1 conditioned media for our endometrial organoid culture media, for which detailed protocols are available. We strongly suggest using homemade conditioned media; however, we provide commercial alternatives in the ‘Reagents’ section.

In our protocol, we maintain primary endometrial epithelial cells as organoids, then dissociate and seed the cells on Matrigel-coated plates, where they attach and proliferate to form confluent monolayers (OFELs) within days of seeding (Fig. 7b-c). It is important to start with an optimal cell number and to initiate the hormonal stimulation only when the monolayer reaches at least 80-90 confluency. Experimenters should expect the cells from different donors to grow at different speeds, thus, we recommend testing the optimal cell seeding number for each donor. Since OFELs and blastoids should be prepared in parallel, the initial cell density should be adjusted so as to obtain blastoids when the layer is confluent. Therefore, some organoid lines may require a modification to the number of seeded cells. OFELs respond to hormonal stimulation by decreasing cell proliferation and upregulating genes that mark the mid-secretory-phase endometrium (Fig. 7d). The experimenter should be aware that outcomes of the hormonal stimulation and of the implantation assay are donor and genetic background dependent.

### Implantation assay

**Fig. 8:**
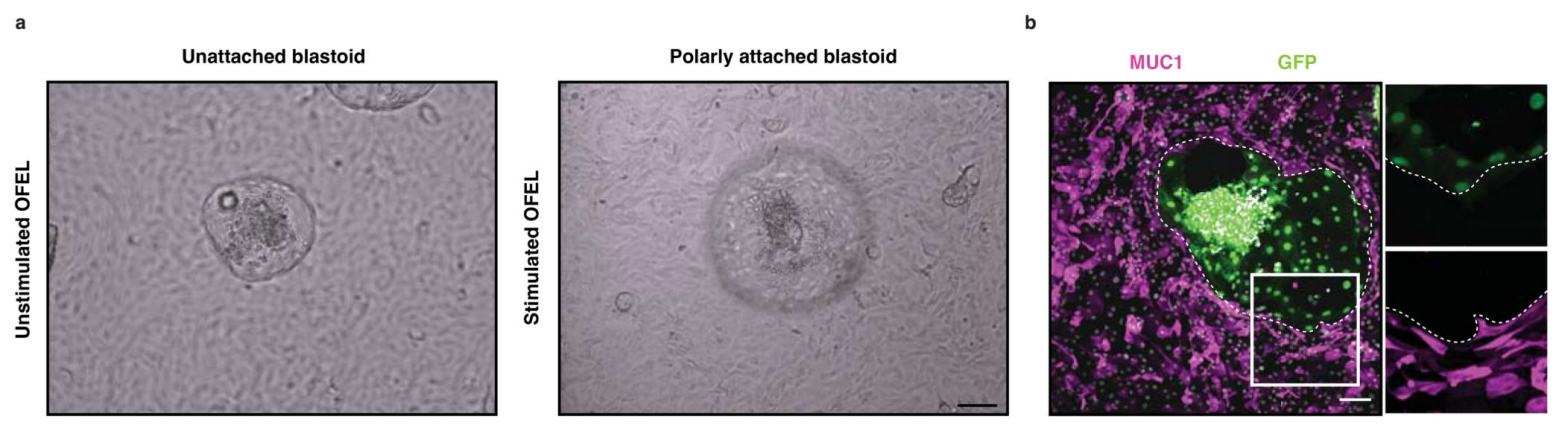
Human blastoids recapitulate aspects of implantation. a, Representative phase-contrast images of blastoids after deposition onto non-stimulated (left) or stimulated (right) OFELs. Human blastoid attached to endometrial cells from the polar region (also see Video 1). b, Immunofluorescence stainings for MUC1 (Magenta), a glycoprotein that highly expresses at the epithelial surface of endometrium in the receptive phase^102^, with an attached GFP+ blastoid. Dashed lines indicate the area that trophoblast cells repelled endometrial cells. Scale bars, 100 μm

It is important to have confluent OFELs and high quality 96 hours blastoids ready for the implantation assay. To do so, blastoid formation must be done in parallel with preparation of OFELs. Blastoid formation (from Steps 35-53) should start 1 day after hormonal stimulation of OFELs (Step 121). Visually inspect the blastoids to assess and record morphology. Only blastoids that display the stringent morphological criteria described in Box 1, including the “hollow-ball” blastocyst morphology and the formation of a small and compact ICM have been tested for their implantation potential (See Fig. 6a). Blastoids can be transfered individually or in a group of 10-15 per well. We recommend transferring 30-50 blastoids per each study group. Blastoids attach to endometrial cells within 24 to 48 hours. This attachment is mediated by the polar region, and this attachment can be quantified by fixing blastoids 36 to 48 hours after deposition and subsequently processed for immunofluorescence staining for blastoids markers such as OCT4 and NR2F2 or by flushing them using a mouth pipette under a microscope (Fig. 8 and Video 1). The percentage of attached structures will report as the percentage of total transferred structures. Alternatively, blastoids formed from GFP+ naive hPSCs can be used for live imaging to trace the direction of attachment and blastoids outgrowth.

## Materials

### Biological materials

Frozen vials of hPSCs. We have successfully formed human blastoids starting from both human embryonic stem cells (hESCs) and human induced PSCs (hiPSCs). Lines that have been used successfully include human hESC lines; WA09 (H9,NIHhESC-10-0062, RRID:CVCL_9773, female), Shef6 (NIHhESC-10-0078, RRID:CVCL_9793, female), CAMe001-A (HNES1, CVCL_9R98, male), hiPSC lines; cR-NCRM2 (RRID:CVCL_1E72, female) and niPSC 16.2.b.

Frozen vials were either obtained in a PXGL state of pluripotency from the laboratories of Austin Smith and Yasuhiro Takashima or in a primed state of pluripotency and then converted to the PXGL state using a chemically defined medium^32^. Conventional primed hPSC can chemically reset to a PXGL state as previously described^96^.

CAUTION Any experimental protocol using hPSCs must comply with national and regional laws and institutional ethical guidelines and regulations. The Austrian Academy of Sciences granted us permission to conduct the experiments presented here.

CRITICAL hPSCs should be regularly authenticated for their state using a SUSD2/CD75^64,92,93^ FACS assay, and checked for mycoplasma contamination.

Frozen vials of human endometrial organoids. We use already established endometrial organoids for OFELs formation and we have successfully formed OFELs from three donors (Supplementary Table 2). Ethical approval from the designated ethical committee at the Royan Institute in Tehran for deriving endometrial organoids and informed consent from patients were both sought when obtaining endometrial tissue (IR.ACECR.ROYAN.REC. 1397.93).

CAUTION Informed consent must be obtained from all subjects. Studies comply with all relevant institutional and governmental regulations.

Mouse Embryonic Fibroblasts (MEFs). E13 embryos from pregnant CF1 mice are used for MEFs production according to the previously published method ^97^. We expand the CF-1 MEFs in MEF medium, irradiate and suspend them in ice-cold cryopreserving solution (usually 1.5–2.0 × 10^6^ cells per ml for one six-well plate) and transfer the cryopreserving tubes to a -80 °C freezer overnight and then the liquid nitrogen tank for long-term storage. We use these cells as feeder cells for hPSCs culture. CF1 MEFs are also commercially-available (Gibco, cat no. A34180, RRID: CVCL_RB05, mixed sex).

CAUTION The use of animals should be approved by the relevant institutional ethics review committees. Our animal experiments are approved by the IMP/IMBA ethical committee and performed in accordance with the guidelines of the institution.

## Reagents

DMEM high glucose (in house) or (Gibco, cat no. 11965-084)

DMEM/F-12 (in house) or (Gibco, cat no. 11320-033)

Neurobasal medium (in house) or (Gibco, cat no. 21103-049)

Advanced DMEM/F12 (Gibco, cat. no. 12634-010)

GlutaMAX, 100x (Gibco, cat. no. 35050-068)

Penicillin-streptomycin (Sigma-Aldrich, cat. no. P0781)

HEPES, 1 M (in house) or (Gibco, cat no. 15630-080)

N2 supplement, 100x (Gibco, cat. no. 17502-048).

CRITICAL Store at −20°C and protect from light as recommended by the manufacturer until the expiration date.

B27 supplement, 50x (Gibco, cat. no. 17504-044).

CRITICAL Store at −20°C and protect from light as recommended by the manufacturer until the expiration date.

Sodium Pyruvate, 100 mM (Sigma-Aldrich, cat. no. S8636)

MEM Non-Essential Amino Acids Solution (NEAA), 100x (Gibco, cat. no. 11140-050)

2-Mercaptoethanol, 50 mM (Gibco, cat. no. 31350-010)

CAUTION 2-Mercaptoethanol is toxic if ingested, inhaled or absorbed through the skin or mucous membranes.

Bovine Serum Albumin solution (BSA), 35% (Sigma-Aldrich, cat. no. A7979-50ML)

Phosphate Buffered Saline solution (PBS) (in house) or (Gibco, cat. no. 10010-023)

Accutase (BioLegend, cat. no. 423201)

TrypLE Express (Gibco, cat. no. 12604-013)

CRITICAL Light sensitive. Store in the dark as recommended by the manufacturer

Anti-Adherence Rinsing Solution (STEMCELL Technologies, cat. no. 07010)

PD0325901 (MedChemExpress, cat. no. HY-10254)

XAV-939 (MedChemExpress, cat. no. HY-15147)

Gö 6983 (MedChemExpress, cat. no. HY-13689)

Human leukemia inhibitory factor (hLIF) (in house) or (Qkine, cat. no. Qk036)

A83-01 (MedChemExpress, cat. no. HY-10432)

1-Oleoyl Lysophosphatidic Acid (LPA) (Tocris, cat. no. 3854)

ROCK inhibitor Y-27632 (MedChemExpress, cat. no. HY-10583)

CRITICAL Y-27632 should be added fresh to the medium.

Nicotinamide (Sigma-Aldrich, cat. no. N0636)

N-acetyl-L-cysteine (Sigma-Aldrich, cat. no. A9165)

Human fibroblast growth factor (FGF)-10 (PeproTech, cat. no. 100-26)

Human FGF-2 (PeproTech, cat. no. 100-18B)

Human epidermal growth factor (EGF) (PeproTech, cat. no. AF-100-15)

Noggin conditioned medium (homemade) or recombinant human Noggin (Peprotech, cat. no. 120-10C)

R-spondin conditioned medium (homemade), or recombinant R-spondin 1 protein (R&D Systems, cat. no. 4645-RS-025)

SB202190 (MedChemExpress, cat. no. HY-10295)

Insulin-Transferrin-Selenium (ITS) (in house) or (Gibco, cat. no. 41400-045)

CMRL Medium, no glutamine (Gibco, cat. no. 21530-027)

Embryonic stem-cell qualified FBS (Gibco, cat. no. 16141-002)

KnockOut Serum Replacement (Gibco, cat no. 10828-028)

β-oestradiol (Sigma-Aldrich, cat, no. E8875)

Progesterone (Sigma-Aldrich, cat. no. P0130)

8-Br-cAMP (Biolog, cat. no. B 007)

ITS-X (Gibco, cat. no. 51500-056)

Sodium lactate (Sigma-Aldrich, cat. no. L7900)

Matrigel, growth factor reduced, Phenol Red-free (Corning, cat. no. 356231)

Geltrex basement membrane matrix (Gibco, cat. no. A1413302)

Gelatin solution (Sigma-Aldrich, cat. no. G1393-100ML)

CryoStor cell cryopreservation media (Sigma-Aldrich, cat. no. C2874-100ML)

Recovery Cell Culture Freezing Medium (Gibco, cat. no. 12648-010)

Paraformaldehyde (PFA; Electron Microscopy Sciences, cat. no. 15710)

CAUTION Formaldehyde is a Group 1 carcinogen classified by the International Agency for Research on Cancer. It should be used in a fume hood and disposed of with precaution. Take safety precautions when handling the PFA..

Triton X-100 (Sigma-Aldrich, cat. no. X100)

Donkey serum (Biowest, cat. no. S2170)

Mouse anti-CDX-2 monoclonal antibody (BioGenex, cat. no. MU392A-5UC; RRID:AB_2650531)

Rabbit anti-NANOG monoclonal antibody (Abcam, cat. no. ab109250; RRID:AB_10863442)

Rat anti-GATA-4 monoclonal antibody (Invitrogen, cat. no. 14-9980-82; RRID:AB_763541)

Rabbit anti-GATA-3 polyclonal antibody (Santa Cruz Biotechnology, cat. no. sc-9009; RRID:AB_640893)

Mouse anti-OCT-3/4 monoclonal antibody (Santa Cruz Biotechnology, cat. no. sc-5279; RRID:AB_628051)

Rat anti-SOX2 monoclonal antibody (Invitrogen, cat. no. 14-9811-80; RRID:AB_11219070)

Rabbit anti-NR2F2 recombinant antibody (Abcam, cat. no. ab211776; RRID:AB_2893028)

Rabbit anti-CDH1 (E-Cadherin) monoclonal antibody (Cell Signaling Technology cat no. 3195S; RRID:AB_2291471)

Goat anti-ZO1 polyclonal antibody (Abcam cat. no. ab190085; RRID:AB_2890613)

Donkey anti-mouse IgG Alexa Fluor 647 (Invitrogen, cat. no. A31571; RRID:AB_162542)

Donkey anti-rabbit IgG Alexa Fluor 568 (Invitrogen, cat. no. A10042; RRID:AB_2534017)

Donkey anti-rat IgG Alexa Fluor 488 (Invitrogen, cat. no. A21208; RRID:AB_2535794)

Donkey anti-goat IgG Alexa Fluor Plus 488 (Invitrogen, cat. no. A32814, RRID:AB_2762838)

Hoechst 33342, Trihydrochloride, Trihydrate, 10 mg/mL solution in water (Invitrogen, cat. no. H3570)

## Equipment

CO2 incubator (Eppendorf, CellXpert C170i)

Biological safety cabinet, SafeFAST Premium 212 (FASTER S.R.L)

AggreWell™ 400 plates (Stemcell Technologies, cat. no. 34415) or Elplasia® plates (Corning, cat. no. 4442) or Gri3D® (Sun Bioscience)

CRITICAL Other commercially available plates that allow controlling aggregate size might be compatible with this protocol.

Falcon tubes, 15 ml (Greiner, cat. no. T1943-1000EA)

Falcon tubes, 50 mL (Thermo Scientific, cat. no. 05-539-13)

Microcentrifuge tubes, 1.5 ml

Microcentrifuge tubes, 0.5 ml

Pipette aid (accu-jet pro, Brand)

5 ml serological pipettes (starlab, cat. no. E4860-0005)

10 ml serological pipettes (starlab, cat. no. E4860-0010)

25 ml serological pipettes (starlab, cat. no. E4860-0025)

P10 micropipette, graduated TipOne Filter Tip, Refills (starlab, cat. no.S1121-2710)

P10/20 micropipette, graduated TipOne Filter Tip, Refills (starlab, cat. no. S1120-3710)

P20 micropipette, graduated TipOne Filter Tip, Refills (starlab, cat. no. S1123-1710)

P100 micropipette, graduated TipOne Filter Tip, Refills (starlab, cat. no. S1123-1840)

P300 micropipette, graduated TipOne Filter Tip, Refills (starlab, cat. no. S1120-9710)

P1000 micropipette, graduated TipOne Filter Tip, Refills (starlab, cat. no. S1122-1730)

Syringe filter, 0.2 µm (vwr, cat. no. CA28145)

Cell strainers, 100 µm (CELLTREAT, cat. no. 229485)

Plates, 6 well (Thermo Scientific, cat. no. 140675)

Plates, 48 well (Eppendorf, cat. no. 0030723112)

CRITICAL It is important to use tissue culture plates that keep the Matrigel droplets spherical, which is critical for 3D organoid culture.

Plate, 96 well (Thermo Scientific, cat. no. 167008)

Glass bottom plate, 96 well (Cellvis, cat. no. P96-1.5H-N) or (Greiner Bio-One, cat. no. 655892P)

µ-Slide Angiogenesis Glass Bottom (ibidi, cat. no. 81507)

Countess II Automated Cell Counter (Invitrogen)

Countess™ II FL Reusable Slide (Invitrogen, A25750)

Hot-Plate (Kunz Instruments, HP-3D, cat. no. 205100)

Centrifuge (5910 R, Refrigerated, Eppendorf)

Inverted microscope (Carl Zeiss MicroImaging, model: Zeiss Observer.Z1)

Scanning confocal microscope

EVOS™ M7000 Imaging System (Invitrogen, cat. no. AMF7000)

EVOS XL Core Imaging System (Invitrogen, cat. no. AMEX1000)

Water bath, Sub Aqua Pro (Grant)

Shaker (vwr, ADV 3500)

Common consumables (serological pipettes, centrifuge tubes, pipette tips, and blades)

## Reagent setup

CRITICAL After thawing the small-molecule stock solutions, they can be stored at 4°C for up to 1 week. Do not repeatedly thaw and freeze the small-molecule stock solutions.

Stock solutions

Stock solutions of all the proteins and chemicals listed in ‘Reagents’ should be prepared and stored following manufacturers’ instructions.

PD0325901

Dissolve 1 mg in 0.2074 ml DMSO to prepare a 10 mM stock solution that can be frozen (−20°C) for up to 1-6 months.

XAV-939

Dissolve 1 mg in 0.3202 ml DMSO to prepare a 10 mM stock solution that can be frozen (−20°C) for up to 1-6 months.

Gö 6983

Dissolve 1 mg in 0.0904 ml DMSO to prepare a 25 mM stock solution that can be frozen (−20°C) for up to 1-6 months.

A83-01

Dissolve 1 mg in 0.2372 ml of DMSO to obtain a 10 mM stock solution that can be frozen (−20°C) for up to 1-6 months.

Y-27632 dihydrochloride

Dissolve 10 mg in 1.561 ml of H2O to prepare a 20 mM stock solution that can be frozen (−20°C) for up to 1-6 months.

CRITICAL Y-27632 should be added fresh to the medium.

1-Oleoyl Lysophosphatidic Acid (LPA)

Dissolve 1 mg in 0.4362 ml of PBS to prepare a 5 mM stock solution that can be frozen (−20°C) for up to 1-6 months.

R-spondin 1 conditioned medium

Make up the medium as described previously^98,99^. Conditioned medium can be stored for 6 months without loss of activity. Use the medium immediately after thawing, and do not freeze it again.

Noggin conditioned medium.

Make up the medium as described previously^99,100^. Conditioned medium can be stored for 6 months without loss of activity. Use the medium immediately after thawing, and do not freeze it again.

Rec. human Noggin

Dissolve 100 μg in 1 ml of PBS + 0.1% (wt/vol) BSA to prepare a 1,000× stock solution that can be frozen (−20°C) for up to 1-6 months.

N-acetyl-L-cysteine

Dissolve 81.5 mg/ml in H2O to prepare a 400× 500 mM stock solution that can be frozen (−20°C) for up to 6 months. Nicotinamide

Dissolve 1.2 g in 10 ml of PBS to prepare a 100× 1M stock solution. Store 0.5 ml aliquots at −20°C until the expiration date.

FGF-10

Dissolve 500 μg in 5 ml of PBS + 0.1% BSA to prepare a 10,000× 0.1 mg/ml stock solution that can be frozen (−20°C) for up to 1-6 months.

Human EGF

Dissolve 1 mg in 2 ml of PBS + 0.1% (wt/vol) BSA to prepare a 10,000× 0.5 mg/ml stock solution that can be frozen (−20°C) for up to 1-6 months.

SB202190

Dissolve 25 mg in 2.75 ml of DMSO to prepare a 30 mM 3,000× stock solution that can be frozen (−20°C) for up to 1-6 months.

FGF2

Dissolve 50 μg in 100 µl of 5 mM Tris, pH 7.6 (0.5 mg/ml). Dilute it to a 10,000× 50 μg/ml stock solution by adding 900 µl of PBS + 0.1% (wt/vol) BSA to 100 µl of 0.5 mg/ml solution that can be frozen (−20°C) for up to 1-6 months.

8-Br-cAMP

Dissolve 100 umol in 100 uL of H2O for a 1 M stock solution that can be stored at 4°C for up to 1-6 months.

Geltrex aliquots

Thaw Geltrex (5 ml vial) on ice in a 4°C fridge overnight and mix well before dividing into small aliquots (60–200 µl) under aseptic conditions. Keep a record of the lot number and protein concentration listed on the product specification sheet. Store aliquots at −20 or −80°C for up to 2 years. Thaw for 2–3 hours on ice in a 4°C fridge before use.

CRITICAL During experiments, Geltrex should be kept constantly on ice.

Matrigel

Thaw the original bottle overnight at 4°C on ice. Mix well by pipetting and divide the Matrigel into 1 ml aliquots in 1 ml cryovials. Aliquots can be stored at −20°C until the expiration date.

CRITICAL During experiments, Matrigel should be kept constantly on ice.

Gelatin solution (0.1% (wt/vol))

Place a vial of 2% (wt/vol) gelatin solution in the water bath at 37°C. Gelatin solutions need to completely liquefy. Once liquified, add 50 mL of the 2% (wt/vol) gelatin solution to 950 mL of PBS or water. Mix the solution and store until the expiration date at 4°C.

MEF medium

To prepare 500 ml of MEF medium, mix 400 ml of DMEM high glucose medium with 100 ml of FBS, 5 ml of GlutaMAX supplement (100×) and 5 ml of NEAA (100×). Store it for up to 4 weeks at 4°C.

N2B27 basal medium

Prepare the medium by adding 1× N2, 1× B27, 1× NEAA, 1× GlutaMAX, HEPES (final concentration 10 mM), Sodium Pyruvate (final concentration 1 mM) and 2-mercaptoethanol (final concentration 100 µM) to DMEM/F12 and Neurobasal media (50/50).

N2B27 basal medium can be kept at 4°C for ≤1 month.

Washing medium

Supplement DMEM/F12 with 0.1% BSA. Store at 4°C for up to a month.

PXGL hPSC medium

To prepare 50 ml of PXGL hPSC medium, add PD0325901 (final concentration 1 µM), XAV-939 (final concentration 2 μM), Gö 6983 (final concentration 2 µM) and human LIF (final concentration 10 ng ml−1) to 50 ml N2B27 basal medium. PXGL medium can be kept at 4°C for a week

Aggregation media

Prepare the medium by adding Y-27632 (final concentration 10 µM) and BSA (0.3 % (wt/vol)) to the N2B27 basal medium. The medium should be made fresh.

PALLY medium

Prepare 1X medium by adding PD0325901 (final concentration 1 µM), A83-01 (final concentration 1 µM), LPA (final concentration 0.5 to 5 µM, need to be optimized based on hPSCs cell line), hLIF (final concentration 10 ng ml-1), Y-27632 (final concentration 10 µM) to N2B27 basal medium. The medium minus LPA and Y-27632 can be made in advance and be kept at 4°C for a week. LPA and Y-27632 must be freshly added to the medium on the day of usage.

2X PALLY medium

Prepare 2X medium by adding PD0325901 (final concentration 2 µM), A83-01 (final concentration 2 µM), LPA (final concentration 1 to 10 µM, need to be optimized based on hPSCs cell line), hLIF (final concentration 20 ng ml-1), Y-27632 (final concentration 20 µM) to N2B27 basal medium. The medium minus LPA and Y-27632 can be made in advance and be kept at 4°C for a week. LPA and Y-27632 must be freshly added to the medium on the day of usage.

Endometrial organoid culture medium

Prepare the medium by adding N2, B27, GlutaMAX and ITS to 1× concentration and N-acetylcysteine (final concentration 1.25 mM), nicotinamide (2.5 mM), EGF (50 ng ml−1), bFGF (2 ng ml−1), HGF (10 ng ml−1), FGF10 (10 ng ml−1), A83-01 (500 nM) and SB202190 (10 μM) to DMEM/F12 supplemented with 10% (vol/vol) Noggin (or 100 ng/ml recombinant Noggin) and 10% (vol/vol) R-spondin 1 conditioned medium (or 500 ng/ml recombinant R-spondin-1). Endometrial organoid culture medium can be kept at 4°C for a week.

Coating plate with Matrigel

Dilute the Matrigel stock solution with cooled DMEM/F12 medium according to the instruction to make a 3% (vol/vol) Matrigel solution. Add 100 µl of Matrigel solution into wells of the 96-well plate, and keep the plate at 37 °C for at least 2-3 h.

EPCX medium

Prepare the medium by adding β-oestradiol (10 nM), progesterone (1µM), 8-Br-cAMP (250 µM) and XAV-939 (10 µM) to the endometrial organoid medium. The medium should be made fresh.

mIVC1

Prepare the medium by adding embryonic stem-cell qualified FBS (20% (vol/vol)), L-glutamine (2 mM), ITS-X (1×), β-oestradiol (8 nM), progesterone (200 ng ml−1), N-acetyl-L-cysteine (25 µM), sodium lactate (0.22% (vol/vol)), sodium pyruvate (1 mM) and Y27632 (10 µM) to advanced DMEM/F12. The medium can be kept at 4°C up to one week

CMRL-1

Prepare the medium by adding Embryonic stem-cell FBS (10% (vol/vol)), L-glutamine (2 mM), N2 (1x) B27 (1x), sodium pyruvate (1 mM), β-oestradiol (8 nM), progesterone (200 ng ml−1) and Y27632 (10 µM) to CMRL medium. The medium can be kept at 4°C for less than a week

CMRL-2

Prepare the medium by adding Embryonic stem-cell FBS (20% (vol/vol)), L-glutamine (2 mM), N2 (1x) B27 (1x), sodium pyruvate (1 mM), β-oestradiol (8 nM), progesterone (200 ng ml−1) and Y27632 (10 µM) to CMRL medium. The medium can be kept at 4°C for less than a week

CMRL-3

Prepare the medium by adding KnockOut Serum Replacement (30% (vol/vol)), L-glutamine (2 mM), N2 (1x) B27 (1x), sodium pyruvate (1 mM), β-oestradiol (8 nM), progesterone (200 ng ml−1) and Y27632 (10 µM) to CMRL medium. The medium can be kept at 4°C for less than a week

Paraformaldehyde (PFA) 4% (vol/vol)

Dilute PFA in PBS to make 4% (vol/vol).

CAUTION Formaldehyde is a Group 1 carcinogen classified by the International Agency for Research on Cancer. It should be used in a fume hood and disposed of with precaution. Take safety precautions when handling the PFA.

PBST solution (0.1% (vol/vol))

Prepare 0.1% (vol/vol) PBST solution by adding 1 ml of Triton-X 100 in 1 liter PBS. Filter through 0.2 µm syringe filter. Store at room temperature (18–22 °C) for up to 6 months.

Permeabilizing buffer (PBST solution 0.3% (vol/vol))

Prepare 0.3% (vol/vol) PBST solution by adding 3 ml of Triton-X 100 in 1 liter PBS. Filter through 0.2 µm syringe filter. Store at room temperature for up to 6 months.

Blocking solution (10% (vol/vol) donkey serum solution)

Add 1 ml donkey serum to 9 mL permeabilization buffer. Filter through 0.2 µm syringe filter. Use fresh reagent or store at room temperature for up to 1 week. Note that the type of serum required is determined by the host of the secondary antibodies.

Primary antibody solution

Dilute primary antibodies in blocking solution to the following final concentrations: anti-CDX-2 (1:100 dilution), anti-NANOG (1:100 dilution), anti-GATA-4 (1:400 dilution), anti-GATA-3 (1:200-300 dilution), anti-OCT-3/4 (1:100 dilution), anti-SOX2 (1:200-400 dilution), anti-NR2F2 (1:100 dilution), anti-CDH1 (1:500 dilution), anti-ZO1 (1:100 dilution)

Secondary antibody solution

Dilute secondary antibodies along with a nuclear dye Hoechst-33342 (1:300) in blocking solution. Use 1:300 dilution for 3D structures and 1:500 dilution for 2D cultures.

Preparation of irradiated mouse embryonic feeder (MEFs) layer

Timing 1 h

CRITICAL Mitotically inactivated MEFs need to be plated at least 1-2 days prior to their use as a feeder layer. It is important to use high-quality feeders.

1. Gelatin coat a 6-well cell culture plate by adding 1 ml 0.1% (wt/vol) Gelatin solution per well.
2. Incubate the plate at 37°C for 30 min.
3. Prepare and prewarm MEF medium at 37°C.
4. Take a vial of the feeder cells from liquid nitrogen and immediately put them into the 37°C water bath for 1-2 min until only a small ice clump is left.
5. Transfer the feeder cells into a 15 ml tube, containing10 ml of pre-warmed MEF medium.
6. Centrifuge the feeder cells at 200 g for 4 min
  CRITICAL the centrifugation is at room temperature unless otherwise stated.
7. Remove the supernatant from the tube, flick the cell pellet several times to disperse and resuspend the feeder cells with 3 ml of the warm MEF medium.
8. Remove the Gelatin solution from the plate and add 1.5 ml of the warm MEF medium to each well.
9. Add 2-3 × 10^5^ cells per well of a 6-well plate (0.5 ml of resuspended cells), and shake the plate to distribute the cell evenly. The final volume per well should be 1.5 to 2 ml per well of a 6-well plate.
10. Culture the cells in a humidified incubator at 37°C with 5% CO2.

CRITICAL STEP Before use, check the quality of the feeder cells. The feeder cells should be growing with a normal MEF morphology and should be confluent. It is strongly recommended to test how long in advance the MEFs should be thawed before plating hPSCs. Inactivated MEFs can be maintained in culture up to a month with occasional medium change.

### Procedure

#### Thawing PXGL human pluripotent stem cells

Timing 30 min

**CRITICAL** Culturing PXGL state hPSCs in 5% O2 is very important for maintaining pluripotency.

CRITICAL At least 1-2 days before thawing or passaging the PXGL hPSCs, prepare a 6-well cell culture plate with irradiated MEF layers.

1. Thaw an aliquot of Geltrex on ice, and dilute fourfold with cold N2B27 basal medium. Keep on ice.
2. Prepare and prewarm washingmedium and PXGL medium at 37 °C.

**CRITICAL** all the cell culture media should be pre-warmed at 37°C before use otherwise stated.

3. Take a cryovial of PXGL hPSCs from liquid nitrogen and immediately put them into the 37°C water bath for 1-2 min until only a small ice clump is left.
4. Bring the cryovial into the biological safety cabinet after spraying the outer surface with 70% (vol/vol) ethanol.
5. Add 1 ml of pre-warmed washing medium to the cryovial and resuspend the cells gently. Immediately transfer the cells into a 15 ml tube containing 10 ml of pre-warmed washing medium.
6. Centrifuge the cells at 200 g for 4 min
7. Remove the supernatant from the tube, flick the cell pellet several times to disperse and resuspend the cells with 1 mlwarm PXGL medium and supplement it with10 µM Y27632.
8. Before seedingthe hPSCs into feeder cells, prepare the plates of MEFs (See Reagent setup) by aspirating the MEF medium and washing the cells with PBS once and add 1.5 ml PXGL medium supplemented with 10 µM Y27632for each well
9. Transfer the hPSCs onto the MEFs at a density of 0.5–1 × 10^4^ cells/cm2. Add 20 µl diluted Geltrex (from step1) per well to the medium, shake the plate to distribute the cells.
10. Culture hPSCs under hypoxic conditions (5% CO2, 5% O2) at 37°C in a humidified environment.
11. Change medium every day with 1.5-2 ml PXGL medium per well.

### Passaging of human PXGL pluripotent stem cells

Timing 1 h

CRITICAL Culturing PXGL hPSCs in 5% O2 is very important for maintaining pluripotency.

CRITICAL At least 1-2 days before thawing or passaging the PXGL hPSCs, prepare a 6-well cell culture plate with irradiated MEF layers (See Reagent setup).

12. Thaw an aliquot of Geltrex on ice, dilute fourfold with cold N2B27 basal medium. Keep on ice.
13. Prepare and prewarm washing medium, PXGL medium, Accutase and PBS
14. Remove the medium from cells and wash with PBS once.
15. Treat the cells with 500 µl Accutase per well of a 6-well plate at 37°C for 5 min.

CRITICAL STEP Treating cells with Accutase for too long could damage the cells.
16. Check the plate under the microscope. Dissociation is completed if the cells become round and some colonies detach after tapping the plate.
17. Use a P1000 pipette to dissociate the colonies into single cells and add 1 ml washing medium to stop dissociation.
18. Collect the cells and transfer them into a 15 ml tube. Centrifuge the cells at 200 g for 4 min.
20. Remove the supernatant from the tube, flick the cell pellet several times to disperse and resuspend the cells with 1 mlwarm PXGL medium and supplement with10 µM Y27632
21. Before seedingthe hPSCs into feeder cells, prepare the plates of fresh MEFs (See Reagent setup) by aspirating the MEF medium,washing the cells with PBS once and adding 1.5 ml PXGL medium supplemented with 10 µM Y27632.
22. Transfer the hPSCs onto the MEFs at a density of 0.5–1 × 10^4^ cells/cm2. If needed add 20 µl diluted Geltrex (from step 12) to the medium per well, shake the plate to distribute the cells.

CRITICAL STEP If the quality of cell culture decreases based on the cell morphology add 20 µl diluted Geltrex per well to the medium during the first 24 h after passaging.

23. Culture hPSCs under hypoxic conditions (5% CO2, 5% O2) at 37°C in a humidified environment.
24. Change medium every day with 1.5 ml PXGL medium per well.

TROUBLESHOOTING

25. Passage PXGL hPSCs every 3–4 d. Otherwise, the number of spontaneously differentiated colonies will increase.

### Blastoid formation in 3 different commercially available plates

CRITICAL It is important to culture PXGL hPSCs for 3 to 4 passages after thawing before starting a blastoid experiment.

#### Preparation of the plates

##### Timing 10-20 min

26. Prepare and pre-warm N2B27 basal medium and aggregation medium.
27. Open plates in a biosafety cabinet.
28. Add 500 µl Anti-Adherence Rinsing Solution to the wells of a AggreWell plate and 200 µl PBS to each well of an Elplasia® 96 well-plate and Gri3D® 96-well plate

CRITICAL STEP Anti-Adherence Rinsing Solution is required during AggreWellpreparation steps to ensure optimal performance. AntiAdherence Rinsing Solution prevents cell adhesion and promotes efficient aggregate formation.

29. Centrifuge the plate at 1300 g for 5 min.

CRITICAL STEP Plates must be well balanced. Prepare a balance plate using a standard plate filled with water to match the weight and position of the plates.

30. Observe the plate under a microscope to ensure the absence of any trapped air bubbles in the microwells. If bubbles remain trapped in any microwells, centrifuge at 1300 g for an additional 5 min.
31. Aspirate Anti-Adherence Rinsing Solution or PBS from the wells.
32. Rinse the wells- of AggreWell plate with 2 ml N2B27 basal medium. Add 100 µl N2B27 basal medium to the wells of an Elplasia 96 well-plate and Gri3D 96-well plate and keep these plates in the incubator at 37°C and continue from step 35 for these plates.
33. Aspirate medium from the wells of the AggreWell plate.
34. Add 250 µl warm aggregation medium to each well of the AggreWell plate to be used and keep the plate in the incubator at 37°C.

#### Formation of hPSC aggregates in the plates

##### Timing 2-3 h

35. Prepare and pre-warm the PXGL medium, N2B27 basal medium, washing medium, PBS and aggregation medium before starting the experiment.
36. For MEF exclusion, prepare a gelatin coated plate by adding 1 ml 0.1% (wt/vol) gelatin into the well of a 6 well plate and incubating at 37°C for more than 30 min.
37. Aspirate the medium from hPSCs after 3 to 4 days culture (from step 25) and wash the cells with 1 ml PBS.
38. Treat the cells with 500 µl Accutase per well of a 6-well plate at 37°C for 5 min.
39. Use a P1000 pipette to dissociate the colonies into single cells
40. Dilute the Accutase with 1ml of washing medium. Collect the cells and transfer them into a 15 ml tube..
41. Centrifuge the cells at 200 g for 4 min.
42. Remove the supernatant and resuspend the pellet in 0.5 ml PXGL medium supplemented with 10 µM Y-27632 for each destination well of the gelatin coated plate. Y-27632 should be added to the medium fresh.

CRITICAL It is important to split the wells 1 in 1 or 2 for MEF exclusion.

43. Remove the gelatin solution from the gelatin coated plate (from step 36) and add 1-1.5 ml PXGL medium supplemented with 10 µM Y-27632 to each well.
44. Add 0.5 ml cell suspension from step 42 to each well for MEF exclusion and incubate the plate at 37°C for 60-90 min.

CRITICAL STEP It is important to exclude all the MEFs and differentiated cells from hPSCs for blastoids formation. MEF can be identified as larger cells as compared to hPSCs.

TROUBLESHOOTING

45. Collect the supernatant containing the unattached hPSCs and transfer into a 15 ml tube
46. Centrifuge the cells at 200 g for 4 min.
47. Aspirate medium and resuspend the cells in 1 ml N2B27 basal medium. Count the cells using countess slides and calculate the volume of medium needed to reach the proper cell number for each well of the plates See supplementary Table 1 for the recommended cell density for various plates.
48. Centrifuge the cells at 200 g for 4 min.
49. Aspirate the medium and add an appropriate amount of N2B27 medium supplemented with 10 µM Y-27632 to have the recommended cell density for the plates. See supplementary Table 1 for the recommended cell density for various plates.

CRITICAL The cell density should be 3.6 to 4 × 10^5^ cells/ml for AggreWell plate, 6 to 6.5 × 10^4^ cells/ml for Elplasia and 3.6 × 10^5^ cells/ml for Gri3D plate.

50. For the H9 cell line, 45 ±10 cells should be seeded per microwell. Therefore, 0.9 to 1 × 10^5^ cells (including surplus considering that some cells fall outside of the microwells) are seeded in 1 well of a 24 well plate, which contains 1200 microwells (Fig. 5a). See supplementary Table 1 for the recommended cell number for seeding for various plates

CRITICAL

It is crucial to prepare a suspension of cells with the appropriate concentration allowing for a specific cell number per well. Initial cell number per well can vary among the different cell lines and need to be optimized.

51. Add 250 µl of cell suspension (1 × 10^5^ cells) to each well of a AggreWells plate (from step 34) and incubate at 37°C and continue from step 53. For the Elplasia and Gri3D plate (from step 32), aspirate medium and add 100 µl of the cell suspension to the wells and continue from step 52. See supplementary Table 1 for the cell suspension volume for various plates. Cells should fall, slide, and gather at the bottom of the wells..

CRITICAL STEP Avoid performing multiple dispensing steps of the cell suspension for each well as this may reduce the accuracy of seeding numbers in each well.

52. Incubate the Elplasia and Gri3D plate in a humidified incubator at 37 °C with 5% CO2 and 5% O2 for 15-20 minutes and then slowly add 100 µl of the pre-warmed aggregation medium.
53. Incubate the cells in a humidified incubator at 37 °C with 5% CO2 and 5% O2 for 24 h.

#### Blastoid development

Timing 3 to 4 days for blastoid formation, 30 min to 1 h each day for changing medium

54. Within 24 h, aggregates of hPSCs can be observed (Day 0) within the wells. Check the aggregate formation under a microscope. Note that all cell lines don’t need this initial aggregagtion step, for example hPSCs from the H9 line can be seeded directly in PALLY medium.

TROUBLESHOOTING

55. To initiate blastoid formation, prepare and pre-warmed 2X PALLY medium for AggreWell plate and Elplasia plate and follow the next steps. Prepare and pre-warmed PALLY medium for Gri3D plate (See supplementary Table 1)

CRITICAL STEP LPA and Y-27632 must be freshly added to the medium on the day of usage.

56. Add 500 µl of pre-warmed 2X PALLY medium to the wells of an AggreWell plate by slowly pipetting down the wall of the wells. For the Elplasia plate, aspirate 100 µl of the medium and add 100 µl of pre-warmed 2X PALLY medium to the wells. For Gri3D plate, aspirate medium and add 200 µl of pre-warmed PALLY medium to the wells. See supplementary Table 1 for the medium and needed volume per well for other plates

CRITICAL STEP Slowly dispensing the medium and gentle moving of the plate helps to prevent displacement of aggregates/blastoids from the microwells.

57. Place the plate into a humidified incubator at 37 °C with 5% CO2 and 5% O2.
58. The next day (day 1), prepare and pre-warmed PALLY medium.
59. Aspirate500 µl of medium of the wells of an AggreWell plate and add 500 µl of pre-warmed PALLY medium to the wells by slowly pipetting down the wall of the wells. For the Elplasia plate, aspirate 150 µl of the medium and add 200 µl of pre-warmed PALLY medium to the wells. For Gri3D plate, aspirate medium and add 200 µl of pre-warmed PALLY medium to the wells.
60. Place the plate into a humidified incubator at 37 °C with 5% CO2 and 5% O2
61. The next day (day 2), check the structure under a microscope.

CRITICAL STEP On day 2 the aggregates should initiate cavity formation.

TROUBLESHOOTING

62. Prepare and pre-warm N2B27 medium supplemented with 500 nM LPA and 10 µM Y-27632.
63. Aspirate 500 µl of PALLY medium and add 500-1000 µl of N2B27 medium supplemented with 500 nM LPA and 10 µM Y-27632 to the wells of an AggreWell plate by slowly pipetting down the wall of the wells. For the Elplasia and Gri3D plates, aspirate 200 µl of the medium and add 200 µl of pre-warmed medium to the wells..

CRITICAL STEP LPA and Y-27632 must be freshly added to the medium on the day of usage.

CRITICAL STEP From day 2 onwards when the majority of aggregates showed cavity formation, medium can be changed with minimal N2B27 medium or clinical IVF medium.

TROUBLESHOOTING

64. The next day, repeat steps 62-63..
65. Blastoid formation is completed on day 4 (Fig 5b and c). Collect aggregates, cavitated structures and blastoids from wells by gently pipetting up and down 2–3 times with a 1-ml pipette. Follow from step 126 for implantation assay and from step 139 for immunostaining.

CRITICAL STEP To minimize the shearing force, cut off the end of the pipette tips before using.

CRITICAL STEP AggreWell plate is a suboptimal platform for blastoid formation: It doesn’t have sufficiently high optical properties to visualize blastoids. In addition, blastoids start floating and fusing after cavitation if the medium is disturbed. This makes media changing difficult on this platform.

CRITICAL STEP Slowly dispensing the medium and gentle moving of the plate helps to prevent displacement of aggregates/blastoids from the microwells.

TROUBLESHOOTING

### Culture and maintenance of human endometrial organoids

CRITICAL We use already established endometrial organoids for OFELs formation. The detailed protocol for generation of endometrial organoids was previously published^72,73^ To restart the cultures after freezing, organoids are carefully thawed, washed, and plated in Matrigel domes.

### Thaw endometrial organoids

Timing 7-10 d cycle for organoid culture, 1-3 h for thawing organoids

66. Preheat 48-well culture plates overnight in a 37°C incubator.

CRITICAL Plates should be preheated overnight by placing them into an incubator at 37 °C. If not properly pre-heated, droplets will flatten out and organoids can easily attach to the bottom of the plate

67. Thaw an appropriate number of Matrigel aliquots in a 4 °C fridge. The needed volume of Matrigel depends on the number of frozen wells in the cryotube. The split ratio after thawing is about 1 in 2. For example, if there are 4 wells of a 48 well plate in the cryotube, thaw around 200 μl Matrigel.

CRITICAL STEP Matrigel needs to be thawed before transferring cryopreserved vials to the water bath.

68. Prepare and pre-warm the endometrial organoid culture medium at room temperature.

CRITICAL STEP Growth factor-containing endometrial organoid culture medium should ideally warm up slowly at room temperature before use. Fast warming up may cause degradation of growth factors.

69. Prepare and pre-warm DMEM/F12 supplemented with10 μM Y-27632 and labeled 15-ml tubes while endometrial organoids are still frozen to minimize thawing and processing times. Delays will lead to a decreased recovery rate.
70. Take a cryovial of endometrial organoids from liquid nitrogen and immediately put them into the 37°C water bath for 1-2 min until only a small ice clump is left.
71. Bring the cryovial into the biological safety cabinet after spraying the outer surface with 7-% ethanol.
72. Add 1 ml of DMEM/F12 containing 10 μM Y-27632 to the cryovial and resuspend the cells gently.
73. Transfer thawed organoid suspension to 15-ml Falcon tubes and add 10 ml DMEM/F12 containing 10 μM Y-27632.
74. Centrifuge 2 min at 400 g. Remove supernatant.
75. Resuspend cell pellet in 100-200 µl Matrigel (based on the number of frozen wells) supplemented with 10 μM Y-27632 using a P200 pipette, be careful to avoid bubble formation. Re-seed each frozen well to 2 wells.

CRITICAL STEP Ensure the suspension is well mixed before proceeding to the next step..

76. Dispense one∼20-µl droplet in a well of a preheated 48-well plate.

CRITICAL STEP It is important to use tissue culture plates that keep the Matrigel droplets spherical, which is important for 3D culture. We recommend using Eppendorf 48-well plates or test the plate for keeping the Matrigel droplets spherical before use.

77. Place the plate upside-down in the incubator at 37 °C and 5% CO2 for 15-20 min to let domes solidify.
78. Add endometrial organoid medium supplemented with 10 μM Y-27632 and keep in the incubator. Refresh medium every 2 days.
79. Culture for 7-10 days, after which organoids can be further expanded (Fig. 7a).

TROUBLESHOOTING

### Endometrial organoid expansion

Timing 7-10 d cycle for organoid culture; 2–3 h for organoid passaging

80. Preheat 48-well culture plates overnight in a 37°C incubator.
81. Check the organoids under a microscope. Estimate the split ratio to use for passaging organoids to calculate the amount of Matrigel to thaw.

CRITICAL STEP Endometrial organoids can be passaged every 7–10 days with a split ratio at 1:3–1:6. The best time to passage endometrial organoids is when most of the organoids reach a diameter around 100 to 200 μm.

82. Thaw an appropriate number of Matrigel aliquots in a 4 °C fridge. 1 ml Matrigel is required for a full 48-well plate of organoids droplets.

CRITICAL STEP Matrigel needs to be thawed before starting the procedure.

83. Prepare and pre-warm the endometrial organoid culture medium and TrypLE Express dissociation medium.

CRITICAL STEP Growth factor-containing culture media should ideally warm up slowly at room temperature before use. Fast warming up may cause degradation of growth factors.

84. Keep DMEM/F12 medium on ice.
85. Remove the culture medium from endometrial organoids.
86. Add 500 ml of ice-cold DMEM/F12 to thewell containing a dome to be harvested. Using a P1000 pre-wetted with DMEM/F12, pipette vigorously to disrupt the Matrigel dome and resuspend the organoids. With the same tip move to another well and disrupt the Matrigel dome and resuspend the organoids.
87. Pool 2-4 wells in a 1.5 ml microcentrifuge tube and centrifuge at 400g for 2 minutes at 4°C.

CRITICAL STEP The first step in passaging organoids is to remove as much Matrigel as possible via mechanical breakup of the domes and washing with a basal medium. The more domes that are processed within a single tube, the more difficult it can be to remove the Matrigel and more washing steps are typically required. We recommend pooling a maximum of 4 wells.

88. Discard the supernatant. Resuspend the organoids in 0.5 ml of pre-warmed (37°C) TrypLE Express dissociation medium supplemented with10 μM Y-27632. Using a P1000, pipette up and down to mix thoroughly. Incubate the organoids at 37°C for 5 - 10 minutes.
89. Add 0.5 DMEM/F12 supplemented with 10 μM Y-27632 and mix organoids.
90. Centrifuge the tube at 400 g for 2 minutes.
91. Carefully aspirate the supernatant without disturbing the pellet and add 150 µl DMEM/F12 supplemented with 10 μM Y-27632.
92. Mix organoids thoroughly by vigorous pipetting to disrupt the organoids as much as possible. Pipetting up and down 80-100x with a P200 pipette. Alternatively, a P10 tip can be placed on top of a P200 tip and used for mechanical disruption of the organoids.
93. Check the organoids using a microscope to ensure sufficient disruption.

TROUBLESHOOTING

CRITICAL STEP Organoids should be dissociated into either individual cells or small fragments. If many whole organoids remain, repeat steps 88-93.

94. Centrifuge fragments at 400g for 2 minutes.
95. Add appropriate amounts of DMEM/F12 supplemented with 10 μM Y-27632 to keep final concentration of Matrigel should be more than 75%-80 (check the next step) and pipetting up and down 10x with a P20 pipette. A split ratio of 1:3–1:6 is typically used.
96. Add ice-cold Matrigel supplemented with Y-27632 and pipetting up and down with a P200 pipette. The final concentration of Matrigel should be more than 75%-80%.

CRITICAL STEP Ensure the suspension is well mixed before proceeding to the next step It is important to avoid formation of air bubbles because they can cause uneven distribution of organoids.

97. Using a P20 pipette, aspirate 20 µl the Matrigel/cell suspension and dispense as small droplets in the middle of the wells of a prewarmed 48-well plate.
98. Repeat for all the remaining Matrigel/cell suspension.

CRITICAL STEP It is important to use tissue culture plates that keep the Matrigel droplets spherical, which is important for 3D culture. We recommend using Eppendorf 48-well plates or test the plate for keeping the Matrigel droplets spherical before use.

99. After seeding all the cells, place the lid on the plate and invert. Place the plate, still inverted, in the cell culture incubator for 15-20 minutes to solidify the Matrigel.
100. While the Matrigel is solidifying, supplement the pre-warmed endometrial organoid culture medium with Y-27632 to a final concentration of 10 µM.
101. After the Matrigel has solidified, add 250 µl of pre-warmed media containing 10 µM Y-27632 per well. Dispense the media along the wall of the well, not directly on the domes.
102. Return the plate to the cell culture incubator. Monitor growth by EVOS microscopy.
103. Perform a complete medium change (without Y-27632) every 2 days.

CRITICAL

Organoids can be passaged or used for OFELs culture every 7-10 days. The split ratio for passaging is around 1:3 to 1:6.

### OFELs Culture

Timing 8-10 d cycle for OFEL culture, 4–6 h for initiation of the culture

CRITICAL Start with organoids in passage (Step 103). Use endometrial organoids after 7-10 days of culture for formation of OFELs.

CRITICAL It is important to start with endometrial organoid cultures in optimal expansion state. The best time to proceed with OFELs formation is when most of the organoids reach a diameter around 200 μm.

CRITICAL STEP It is crucial to have both confluent OFELs and blastoids ready for the implantation assay. To do so, blastoid formation (from step 26) must be done in parallel with preparation of OFELs after 1-day E2 treatment (step 122).

104. Before dissociation of organoids, coat a 96-well cell culture plate with 3% Matrigel for at least 2-3 hours (See reagent setup).
105. Prepare and pre-warm endometrial organoid culture medium.

CRITICAL

Growth factor-containing culture media should ideally warm up slowly at room temperature before use. Fast warming up may cause degradation of growth factors.

106. Pre-warm TrypLE Express dissociation medium.
107. Keep DMEM/F12 medium on ice.
108. Remove the culture medium from endometrial organoid cultures.
109. Add 0.500 ml of ice-cold DMEM/F12 to each well containing a dome to be harvested. Using a P1000 pre-wetted with DMEM/F12, pipette to disrupt the Matrigel® dome and resuspend the organoids. With the same tip move to another well and disrupt the Matrigel dome and resuspend the organoids.
110. Pool 2-4 wells in a 1.5 ml microcentrifuge tube and centrifuge at 400g for 2 minutes at 4°C. Discard the supernatant.

CRITICAL STEP During this step, undissolved Matrigel often accumulates over the organoid pellet. It is important to remove as much Matrigel as possible without removing the organoids. If this is not possible, resuspend the pellet in cold DMEM/F12 and incubate on ice for 10 min, and then repeat the previous step.

111. Resuspend the organoids in 0.5 ml of pre-warmed (37°C) TrypLE supplemented with 10 μM Y-27632. Incubate the organoids at 37°C for 5-7 minutes. (Up and down or knock the tube every 2-3 min)
112. Add 0.500 ml of ice-cold DMEM/F12 to each tube and centrifuge at 400g for 2 minutes at 4°C.
113. Discard the supernatant. Resuspend the organoids in 150-µl DMEM/F12 supplemented with 10 μM Y-27632 and use a P200 pipette to break the organoids to single cells by pipetting up and down 50-100 times. Alternatively, a P10 tip can be placed on top of a P200 tip and used for mechanical disruption of the organoids.

TROUBLESHOOTING

CRITICAL STEP Check progress regularly under a microscope, and stop once the majority of fragments consist of single cells.

114. Add 1 ml DMEM/F12 supplemented with 10 µM Y-27632and pass the digest through a 40-70 μm nylon mesh cell strainer.
115. Centrifuge at 400g for 4 minutes at 4°C. Aspirate and discard the supernatant.
116. Resuspend the cell pellet in endometrial organoid culture medium supplemented with 10 µMY-27632.
117. Take a 15 µl aliquot of the cell suspension to determine cell density.

CRITICAL STEP Ensure the suspension is well mixed before taking the aliquot.

118. Add 15 µl of Trypan Blue to the 15 µl cell suspension and mix well. Count cells by adding 10 µl of the suspension to each side of a disposable Countess chamber slide. Determine cell density from both sides of the slide, and record live cell density and % viability.

CRITICAL STEP If there is a large discrepancy between the two cell counts, repeat cell counting with a new aliquot.

TROUBLESHOOTING

119. Calculate the total number of cells needed from the cell suspension to seed the desired number of monolayers. For 96-well plate seed 2.5-3,5 × 10^4^ cells per well.

CRITICAL STEP It is important to start with an optimal cell number. Although we did not test many donors for OFEL formation, we observed a variability in the successful rate of OFEL formation from donors tested. For example, the optimum cell number for donor 1 was 2.5 × 10^4^ and this was 3.5 × 10^4^ for donor 2. We recommend testing the optimal cell number for each donor before starting implantation assay. Low or high cell density results in an asynchronous experiment. There is donor-to-donor variability in monolayer formation and remaining intact monolayer after stimulation.

120. Culture with the organoid medium for 1 to 2 days to reach more than 80 to 90% confluency (Fig 7b).

TROUBLESHOOTING

### Hormonal stimulation

Timing 6 d cycle of hormonal stimulation, 0.5-1 h changing media

121. Prepare endometrial organoid culture medium and supplement it with 10nM E2.

CRITICAL STEP Medium supplemented with hormones should be freshly prepared.

122. Replace the medium with endometrial organoid culture medium supplemented with 10nM E2. Culture for 2 days. Change the medium every day.
123. Prepare EPCX medium according to ‘Reagent setup’ or include different factors if testing their effect on implantation.
124. Replace the media with EPCX medium to treat the cells with the different hormonal stimulation factors. Culture for 4 days, change the medium every day.

CRITICAL STEP Dead/detached cells are expected to be floating on top of the monolayer. It is important to remove these cells to keep culture more stable.

125. Return the plate to the incubator and check the OFELs under an inverted microscope every day to inspect the confluency. After 4 days, the OFELs are ready for an Implantation assay and the organoids are ready for preparing OFELs (Fig. 7b).

CRITICAL STEP OFELs should be confluent before blastoid deposition (Fig 7b and c). TROUBLESHOOTING

### Implantation assay

#### Blastoid picking for implantation assay

Timing up to 1-3 h

126. Collect aggregates, cavitated structures and blastoids from AggreWells (from step 65) by gently pipetting up and down 2–3 times with a 1-ml pipette.

CRITICAL STEP To minimize the shearing force, cut off the end of the pipette tips before using.

127. Transfer all the structures into wells of a 24-well flat bottom ultra-low attachment plate containing 0.5 ml N2B27 basal medium.
128. Using a stereomicroscope, visually identify intact and good morphology blastoids and transfer them into a separate well of a 24-well flat bottom ultra-low attachment plate containing 0.5 ml CMRL-1 basal medium

CRITICAL STEP Keep the plates on a heating stage (37 °C) in the whole procedure.

129. Place the plate in the incubator and proceed to the next steps.

### Blastoids transfer onto OFELs

Timing up to 3-4 h

130. Prepare CMRL1 medium according to ‘Reagent setup’. mIVC1 medium can also be used for the first 2 days as an alternative.
131. Remove the media from OFELs (step 125) and wash carefully with 100 µl warmed DMEM/F12 twice.

CRITICAL STEP Do this very gently, otherwise the cells detached from the surface.

132. Remove DMEM/F12 from OFELs and add 100 µl media CMRL1 to OFELs 2 hours before blastoids transfer.
133. Using a stereomicroscope with a heating stage visually inspect the blastoids to assess and record morphology. Keep the plates on a heating stage in the whole procedure.

CRITICAL STEP Only blastoids that display the classic blastocyst morphology with compact ICM have implantation potential (See Fig. 6a).

134. Transfer the blastoids with good morphology and onto the OFELs. Blastoids can be transferred individually or in a group of 10-15 per well. We recommend transferring 30-50 blastoids in total in 3 to 5 wells per each study group.

CRITICAL STEP Only transfer blastoids onto fully confluent OFELs.

135. Place the plate in the incubator and incubate overnight.
136. The next day, visually inspect the blastoids under a microscope and test attachment by flushing with a mouth pipette in one well If the blastoids are attached, Add 100 µl of pre-equilibrated CMRL-1 medium, otherwise proceed to the next day. Human blastoids start to attach to endometrial cells within 24 to 48 hours.

TROUBLESHOOTING

137. Calculate attachment efficiency 36 to 48 hours after deposition., Remove medium, wash the wells with PBS and fix using 4% PFA for 30 minutes at room temperature, wash samples three times with PBS for 10 min, and subsequently process for immunofluorescence staining (step 145). Blastoids that did not attach to the endometrial cells, remain floating and can wash out from the well, however, the attached blastoids repel the endometrial cells and remain in the well after washing steps. Alternatively, flush the blastoids using a mouth pipette under a microscope (See Video 1 and 2). The percentage of attached structures are reported as the percentage of total transferred blastoids.
138. In case of need for an extended culture, change half of the media with CMRL-2 supplemented with 5% Matrigel on day2 and half of the media with CMRL-3 supplemented with 5% Matrigel on day3.

### Immunofluorescence staining

CRITICAL Throughout the protocol, aggregates, cavitated structures and blastoids can be fixed and immunostained.

139. Collect aggregates, cavitated structures and blastoids from AggreWells by gently pipetting up and down 2–3 times with a 1-ml pipette.

CRITICAL STEP To minimize the shearing force, cut off the end of the pipette tips before using.

140. Transfer all of the structures into a well of a 24-well flat bottom ultra-low attachment plate containing 0.5 ml N2B27 medium.
141. Keep the plate on a shaker at 50 rpm for 5 min.
142. When all of the structure settled down, remove media carefully under a stereomicroscope and add fixative solution (PFA 4%) and fix samples for 30 min at room temperature.

CAUTION Formaldehyde is a Group 1 carcinogen classified by the International Agency for Research on Cancer. It should be used in a fume hood and disposed of with precaution.

143. Remove PFA and wash samples three times with 1 ml PBS for 10 min. Keep the plate on a shaker at 50 rpm.
144. Select good morphology blastoids and transfer them into 96-well ultra-low attachment plates for the following steps.
145. Permeabilize the structures with 100 µl of the permeabilization solution for 30 min at room temperature on a shaker at 50 rpm.
146. Block the structures to prevent nonspecific binding with 100 µl of the blocking solution for at least 2 h at room temperature on a shaker at 50 rpm.
147. Prepare a primary antibody solution (see ‘Reagent setup’). Remove blocking solution and add100 µl of the primary antibody solution and incubate samples overnight at 4°C on a shaker at 50 rpm.
148. Wash samples three times with PBST 0.1% for at least 10 min. Keep the plate on a shaker at 50 rpm.
149. Prepare a secondary antibody solution (see ‘Reagent setup’). Add 100 µl of secondary antibody solution together with Hoechst for nuclear staining.

CRITICAL STEP From this step onward, the samples must be covered by aluminum foil to avoid photobleaching.

150. Incubate samples for 1 h at room temperature on a shaker at 50 rpm.
151. Wash samples three with PBST 0.1% for at least 10 min. Keep the plate on a shaker at 50 rpm.
152. For blastoid imaging, transfer the samples into the glass bottom µ-slide or a glass bottom plate in the mounting media. The mounting medium should be selected based on the objective used for the imaging. CRITICAL STEP When imaging, it is important to match the refractive index of the mounting medium with the refractive index of the imaging objective’s immersion medium. For example, use PBS or other aqueous buffers for water immersion objectives. Avoid using medium containing phenol red.
153. Perform fluorescence imaging using a confocal microscope.

### Troubleshooting

Troubleshooting advice can be found in Table 1.

### Timing

Steps 1-11, Thawing PXGL naive hPSCs: 30 min for seeding

Steps 12-25, Passaging and culturing naive hPSCs: 30 min to 1hBox 2, Cryopreserving PXGL naive hPSCs: 30 min

Steps 26-34, Preparation of the plates for blastoid formation: 10-20 min

Steps 35-53, Formation of hPSC aggregates in the plates: 2-3 h

Steps 54-65, Blastoid development; 3 to 4 days for blastoid formation: 30 min to 1 h each day for changing medium

Steps 66-103, Culture and maintenance of human endometrial organoids: 7-10 d cycle for organoid culture; 2–3 h for organoid passaging

Box 4, Cryopreserving endometrial organoids: 30 min

Steps 1044-125, OFELs formation and hormonal stimulation; variable; up to 8-10 days for whole process; 30-1 h every day for changing medium

Steps 126-138, Implantation assay; up to 5h

Downstream assays: variable

### Anticipated results

Fig. 4 shows representative images of hPSCs cultured in PXGL. Optimal culture consists of homogeneous round-shaped domed colonies. Extended DataFig. 1 shows representative images of blastoids at different time points of formation. When hPSCs are seeded onto microwell plates, small aggregates form within 1 day, and increase in size each day as shown in Extended DataFig. 1. Using (i) optimal cell number, (ii) triple inhibition of the Hippo, ERK and TGFβ pathways, (iii) adjusted concentration of components (especially LPA), and (iv) adjusted duration of the triple inhibition, we observe that 70–80% of all aggregates display a blastocyst-like morphology (see Box 1) within 3-4 days (Fig. 5 a-d). Across multiple species, the acquisition of an apical domain including PKC, and the inhibition of the Hippo pathway are critical to the formation of TE ^2^. Accordingly, we have shown that ligands of the LPA that inhibit the Hippo pathway enhanced the formation of blastoids (Fig. 5d).

Once established, human blastoids can be identified by light microscopy (Fig. 5 and 6a) and immunofluorescence staining. Fig. 6b and 6c show representative images of blastoids when stained for three lineage markers and NR2F2 as a marker for polar TE.

Over 4 days, the cell number and overall size of the aggregates increases in a range similar to day 5-7 human blastocysts (morphological stages B3-B6). The TE cellular analogues (GATA2+GATA3+CDX2+TROP2+) form within 1 day after induction with PALLY. Depending on the hPSC line used, small cavities become apparent around 2 days after induction with PALLY. After 3-4 days, the polar TE analogues mature as marked by upregulation of NR2F2 and downregulation of CDX2 (Fig. 6c). All blastoids form a compactinner cluster of cells comprising analogs of the EPI (IFITM1High/PRDM14High/ARGFXHigh/TDGF1High/DPPA4High/DNMT3LHigh/SUSD2High)^101^, and of the PrE (GATA4+) lineages (see https://petropoulos-lanner-labs.clintec.ki.se/). It is important for blastoids to be only composed of analogs of the blastocyst-stage cells. Here, single cell transcriptomics analysis of blastoids shows that structures form only three main transcriptomic states marked by genes specific to the EPI, PrE, TE. Moreover, comparison with cells from blastocysts, *in vitro* cultured blastocysts, and a gastrulation-stage embryo confirm the transcriptional similarity of blastoids to the blastocyst stage and dissimilarity from post-implantation ones^63,64^.

Regarding the maintenance of endometrial organoids and the preparation of open faced monolayers from these organoids, Fig. 7a shows representative images of endometrial organoids over time during organoid culture. During the 3D expansion of the organoids, their size and number should increase over time, while maintaining a cyst-like morphology. Fig. 7b shows representative images of OFELs over time. Monolayers start to form 1-2 days after seeding and should keep the integrity after stimulation Fig. 7c shows representative images of OFELs stained for ZO-1 and E-cadherin to check the integrity of OFELs after hormonal stimulation. Upon plating in 2D, OFELs respond to hormones (E2, P4) and to Wnt inhibition by regulating the expression of genes specific to the mid-secretory phase of endometrium (*e.g*., PAEP, LIF, SPP1, GPX3, DPP4) (Fig. 7d).

When human blastoids are deposited onto stimulated OFEL, they attach to and repel endometrial cells within 24 to 48 hours (Fig. 8). This attachment is mediated with the polar region, which can be quantified by fixing blastoids 36 to 48 hours after seeding or by flushing them after 48 hours followed by counting the percentage of attached blastoids. In contrast, human blastoids deposited onto non-stimulated OFEL do not attach as long as the layer is confluent. Fig. 8a shows representative images of unattached blastoids on non-stimulated OFELs and polarly attached blastoids on stimulated organoids (See also Video 1 and 2). We included the representative image of blastoids that did not attach to the cells and remained floating (Fig. 8a, right, See Video 2); and of blastoids that attached and repelled the endometrial cells (Fig. 8b, c). The level of maturation of blastoids is also important for attachment as early blastoids attach less or not at all. The maturation speed of blastoids can vary from line to line and also overtime using the same cell line. Blastoids should thus be regularly and precisely evaluated by morphology (*e.g*., time of cavity formation) and by staining (*e.g*., NR2F2+ polar region). Upon extended culture after implantation assay or on Matrigel coated plate, the three lineages consistently expand and attain several features of post-implantation human embryos. The trophoblasts of blastoids differentiated into SCT and EVT expressing CGβ and HLA-G, respectively. Overall, we concluded that blastoids are capable of recapitulating aspects of implantation and post-implantation development.

## Reporting Summary

Further information on research design is available in the Nature Research Reporting Summary linked to this article.

## Extended Data

**Extended Data Fig. 2:**
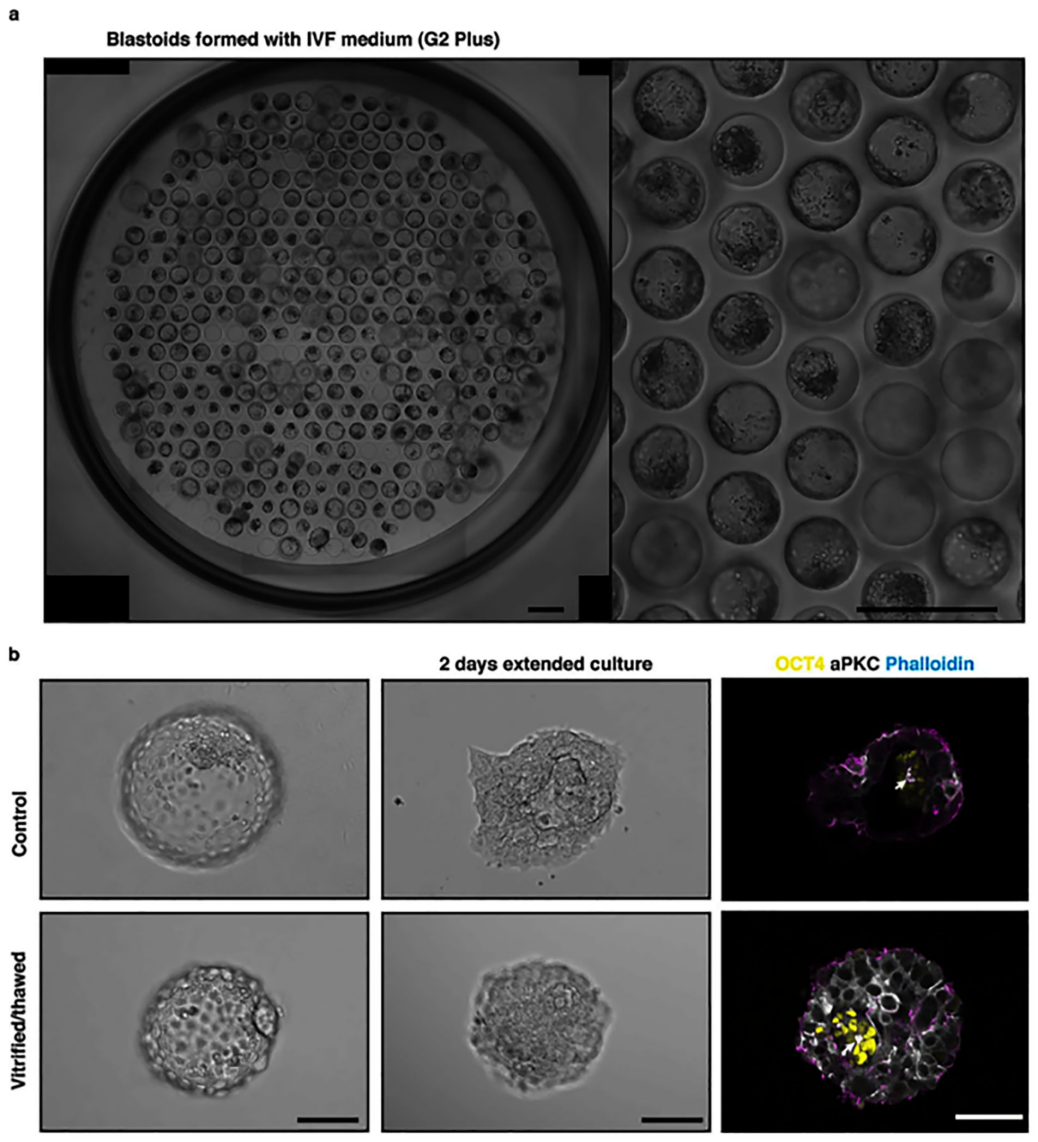
Human blastoids formation in IVF medium and vitrification. a, Bright-field image of human blastoids formed after 48 h stimulation with PALLY medium followed by the use of IVF medium (G2, Vitrolife) for the last 2 d. Scale bars, 400 μm. b, Bright-field image of control (top) and vitrified-thawed human blastoid (bottom) and after 2 d extended culture on Matrigel-coated plate. Scale bars, 100 μm. c, Confocal immunofluorescence image of OCT4 (yellow) and aPKC (gray) in control (top) and vitrified-thawed human blastoid (bottom) cultured on Matrigel-coated plate for 2 d, counterstained with phalloidin marking F-actin (cyan). Arrows point to the pro-amniotic-like cavity. Scale bar, 100 μm.,

## Supplementary Material

Supplementary Materials

Supplementary Video Legends

## Figures and Tables

**Table 1:** Troubleshooting table.

Step	Problem	Possible reason	Solution
24	Substantial, spontaneous differentiation of hPSCs	hPSCs do not react to mouse LIF.	Use human LIF.
		Mycoplasma contamination.	Check whether the cells and media are contaminated with Mycoplasma. Discard contaminated cultures.
		The small molecules are inactive or present in the wrong concentration.	Use fresh small molecules (stored less than 1 week at 4°C, or six months at – 20°C).
		Need for adding Geltrex	Add Geltrex (0.5 µl/cm^2^) to the medium during the first 24 hours after each passage.
44	MEFs are present after the exclusion	Gelatin coating is not properly performed	Increase time for coating
		Time for exclusion is not enough	Increase the exclusion time
54	Most of the hPSCs are dead after seeding	The Y-27632 is inactive.	Store Y27632 in the dark at −20°C for up to 6 months or at 4°C for up to 1 week, respectively. Add fresh to the medium at time of seeding.
		Aggregate formation is not optimal	Check the quality of the plates
61	Low blastoid formation efficiency	LPA is inactive or at suboptimal concentration.	Store LPA in the dark at −20°C for up to 6 months or at 4°C for up to 1 week, respectively. Add fresh LPA to the medium. Increase LPA concentration up to 5 µM.
		Duration of blastoid induction is not sufficient	Increase time of PALLY treatment
		Cavitated structures collapse or don’t expand	Change medium to N2B27 medium after seeing cavities in the majority of structures (from day 2 onwards)
65	Blastoids start floating and fusing after cavitation	AggreWell plates are suboptimal platform for blastoid formation	Slowly dispense the medium to prevent displacement of aggregates/blastoids from the microwells. Use the alternative plates
65	Blastoids size is big or small	Inadequate cell number seeded.	Vary the number of seeded cells and adjust the plating density according to the used platform and cell line Perform a pilot experiment and adjust cell number for your cell line by checking blastoid efficiency and blastoid size.
79	Poor growth of organoids	Poor quality of conditioned medium.	Re-prepare conditioned medium.
		The small molecules and growth factors are inactive or present in the wrong concentration.	Use fresh small molecules and growth factors (stored less than 1 week at 4°C, or six months at –20°C).
		Donor-to-donor variability	Perform the experiment with endometrial organoids from different donors
93, 113	Presence of large cell clusters	Insufficient dissociation of the organoids.	Tap the tube every 1 min during TrypLE treatment Repeat enzymatic and mechanical dissociation.
119	Cell number is less than expected	Endometrial organoids my not be healthy	Check if the organoids cultured in Matrigel grow well.
120, 125	No confluent monolayer forms	Poor cell state upon seeding.	Start with organoid cultures in optimal expansion state. We recommend moving to subsequent stages of the procedure when organoids reach the indicated size (typically around 200 µm) rather than at a particular time Include ROCK inhibitor Y27632 from the time of dissociation onward.
		Inadequate cell number seeded.	Vary the number of seeded cells and adjust the plating density according to the growth rate of the organoid line
		Suboptimal culture condition.	Use the fresh small molecules and growth factors.
		Matrigel coating is not properly performed	Check the Matrigel quality and wash unbound Matrigel gently. Increase time for Matrigel coating.
		Donor-to-donor variability	Do not initiate stimulation until the monolayer is 80-90% confluence.
			Donor-to-donor variability can affect successful establishment of monolayers. Perform a pilot experiment with endometrial organoids from different donors to test monolayer formation and stability after hormonal stimulation.
		High passage number	We recommend using organoids at low passage (less than 10)
136	No blastoid attachment	Endometrial cells did not respond to hormonal stimulation	Use the fresh small molecules, growth factors and hormones
		Donor-to-donor variability	Perform the experiment with endometrial organoids from different donors
		Inadequate cell number seeded.	High cell density results in an asynchronous experiment, start with an optimal cell number.
		Blastoids with low quality or wrong stage	Check blastoid maturation by staining (*e.g.*, NR2F2+ polar region) Have control wells without endometrial cells to check blastoid attachment and outgrowth

## Data Availability

The main data discussed in this protocol were generated as part of the study published in the supporting primary research papers by Kagawa et al^61^. Source data are provided with this paper. Representative results obtained using this protocol are available within the article, with additional examples available from the corresponding author upon request.

## Box 1 Recommended methods for characterizing human blastoids

Morphological analysis to monitor the pace of the growth and inflation of blastoids

Morphologically, a fully-formed human blastoid should display the classic hollow-ball blastocyst morphology with a thin, circular monolayer of outer cells, a compact cluster of inner cells, and an overall diameter more than 180 μm^88^. Although blastoids and blastocysts undergo cycles of deflation/inflation that decrease their overall size, this does not seem to affect a high percentage of blastoids at a given time. As such, considering smaller cavitated structures as blastoids on day 4 is likely to include aberrant structures not conforming to the stringent criteria. See Fig. 6 for an example of a typical blastoid and Fig. 3 for proposed inclusive criteria.

Immunostainings to monitor the sequential formation of the analogues of the three lineages

Human blastoids should consist of analogues of the three founding lineages that form in accordance with the sequence seen in human development. TE and EPI should form first (24-65 hours), while pTE and PrE should form second (65-96 hours). TE cells can be identified by immunofluorescence using GATA2, GATA3, CDX2 and TROP2 antibodies. EPI can be identified by immunofluorescence using OCT4, NANOG antibodies, and PrE using GATA4, SOX17 and PDGFRa antibodies. Polar TE analogues can be distinguished by the upregulated expression of NR2F2 and CCR7, and the downregulated expression of CDX2. See Fig. 6b,c for an example of blastoids immunostained for the three lineages.

(sc)RNA-seq analysis to monitor the transcriptomic similarity to the blastocyst-stage cells

The predictive power of blastoids depends on whether they are composed of analogs of the blastocyst-stage cell types and devoid of post-implantation derivatives, or of other off-target cell types. The states of blastoid cells can be evaluated through scRNAseq analysis. Their transcriptional similarity to specific embryonic stages can be measured using blastoid cells alone and by comparison with primed hPSCs or hTSCs that reflect post-implantation stages. More importantly, the transcriptomic state of blastoid cells can be evaluated by projecting them in silico on top of reference maps formed using cells isolated from embryos at different stages. This allows their transcriptomic state and the stage equivalence to be inferred^64^. We have described this method in another publication^49^.

Implantation and post-implantation assays to monitor the axis formation and differentiation potential

The human blastocyst initiates implantation through the attachment of the pTE, which is defined by its contact with the EPI, to a receptive endometrium. Human blastoids should be able to model this interaction with endometrial organoid cells. See Figure 8 and Video 1 for examples of attached blastoids. Upon prolonged culture after implantation assay (up to 4 days) or extended culture on Matrigel coated plates (up to 6 days), the three lineages of blastoids should consistently expand and form pro-amniotic-like cavities enriched with F-actin, PODXL and aPKC. Trophoblast cells should be able to differentiate into SCT and EVT expressing CGβ and HLA-G respectively.

Additional methods that can be used to characterize human blastoids

In future, analysis of transposon expression, DNA methylation, and of the X chromosome status could provide landmarks to refine the evaluation of the developmental stages.

**[PRODUCTION: END OF BOX 1]**

## Box 2 Cryopreserving PXGL human pluripotent stem cells

Timing 30 min

CRITICAL

For PXGL hPSCs grown in 6-well plates half to one well of colonies at 70-80% confluency can be frozen in one cryovial.

1. Prepare and prewarm washing medium, PXGL culture media, Accutase and PBS.
2. Prepare labeled cryovials. We recommend freezing 2-3 vials per each well.
3. Remove the medium and wash cells with PBS once.
4. Treat the cells with 500 µl Accutase per well of a 6-well plate at 37°C for 5 min.

CRITICAL STEP Treating cells with Accutase for too long could damage the cells.

5. Check the plate under the microscope. Dissociation is completed if the cells become round and some colonies detach after tapping the plate.
6. Use a P1000 pipette to dissociate the colonies into single cells and add 1 ml washing medium to stop dissociation.
7. Collect the cells and transfer them into a 15 ml tube.
8. Centrifuge the cells at 200 g for 4 min.
9. Remove the supernatant from the tube, flick the cell pellet several times to disperse and resuspend the cells in 500 µl of cold freezing medium (CryoStor CS10) per a cryovial.

CRITICAL STEP Freezing medium should be stored at 4°C and can be used immediately once taken out.

10. Transfer 500 µl of cell suspension to a cryovial and immediately place cryovials in a Mr. Frosty container.
46. Transfer the container in a -80°C freezer.

CRITICAL STEP Do not keep the cryovials in the -80°C freezer for a long term.

11. Transfer the cryovials into a liquid nitrogen tank.
  CAUTION

Wear protective gloves and a face mask while handling liquid nitrogen

PAUSE POINT

Cells can be frozen in liquid nitrogen for several years.

## Box 3 Blastoid vitrification and thawing

After Step 66, blastoid vitrification can be performed.

**Materials specific to blastoid vitrification/thawing**

Vitrification Media (Kitazato, VT801, 91171)

Thawing Media (Kitazato, VT802, 91182)

Cryotop (Kitazato)

Repro plate (Kitazato, 83006)

***Human blastoid vitrification***

1. Using a stereomicroscope with a heating stage visually inspect human blastoids from step to assess and record morphology.
2. Select only human blastoids that display the classic blastocyst morphology with a compact ICM.
3. Follow the instruction of the vitrification-cryopreservation protocol. Briefly, equilibrate human blastoids at room temperature for 12–15 min in an equilibration solution (ES). Then immerse human blastoids in vitrification solution (VS) and transfer blastoids to the vitrification device (Cryotop) at 1 min after immersion in the vitrification solution. Then, remove surplus vitrification solution, and directly place the device in liquid nitrogen for rapid freezing.
4. Transfer the vitrification device to a LN2 tank for long storage.

***Human blastoid thawing and extended culture***

1. Prewarm thawing solution (TS) at 37 °C for 2 hours.
2. Prepare and equilibrate CMRL-1 medium at 37 °C for 2 hours.
3. Prepare a Matrigel coated plate.
4. Add 100 μl CMRL-1 medium supplemented with 10 μM Y-27632 to the wells of the Matrigel coated plate and keep the plate in the incubator.
4. Follow the instruction of the thawing protocol. Briefly, remove the vitrification device containing human blastoids from liquid nitrogen and transfer to a thawing solution heated at 37 °C. Find human blastoids in the medium, after 1 min, gently aspirate human blastoids and transfer them into a dilution solution. Wash human blastoids twice in washing solution and follow the next step for extended culture.
5. Wash blastoids with CMRL-1 medium supplemented with 10 μM Y-27632.
6. Transfer blastoids in the wells of the Matrigel coated plate.
7. Keep the plate in a humidified incubator at 37°C with 5% CO2.
8. Next day, add 5 % Matrigel to CMRL-1 medium, supplement medium with 10 μM Y-27632.
9. Equilibrate medium at 37 °C for 2 hours.
10. Add 100 μl CMRL-1 medium to each well and keep the plate in the incubator.
11. Next day, fix extended cultured blastoids using 4% PFA for 30 minutes at room temperature, wash samples three times with PBS for 10 min, and subsequently process for IF staining.

The blastoid morphology was stable after vitrification-thaw cycle (Extended Fig. 2b), and upon extended culture, control and vitrified-thawed blastoids maintained expression of OCT4 and formed pro-amniotic-like cavities enriched with aPKC and F-actin (Extended Fig. 2b, c).

## Box 4 Cryopreserving endometrial organoids

Timing 30 min

CRITICAL It is important to start with endometrial organoids after 5-7 days of culture. About 3-5 matrigel droplets full of endometrial organoids is sufficient for one cryovial.

1. Remove the culture medium.
2. Use 0.500 ml of ice-cold DMEM/F12 for 2-4 wells containing a dome to be harvested. Using a pre-coated P1000, pipette to disrupt the Matrigel® dome and resuspend the organoids, move to another well, disrupt the Matrigel® dome and resuspend the organoids.
3. Pool 2-4 wells in a 1.5 ml tube and centrifuge at 400g for 2 minutes at 4°C.
4. Discard the supernatant. Resuspend the organoids in 0.5 ml of pre-warmed (37°C) TrypLE supplemented with 10 μM Y-27632. Incubate the organoids at 37°C for 5-7 minutes. (Up and down or knock the tube every 2-3 min)

CRITICAL STEP During centrifugation, undissolved Matrigel often accumulates over the organoid pellet. It is important to remove as much Matrigel as possible without removing the organoids. If this is not possible, resuspend the pellet in cold DMEM/F12 and incubate on ice for 10 min, and then repeat the previous step.

5. Add 0.500 ml of ice-cold DMEM/F12 to each tube and centrifuge at 400g for 2 minutes at 4°C.
6. Discard the supernatant. Resuspend the organoids in 500 µl of cold freezing medium (Recovery Cell Culture Freezing Medium containing 10 μM Y-27632) per a cryovial.

CRITICAL STEP Freezing medium should be stored at 4°C and can be used immediately once taken out.

7. Transfer 500 µl of cell suspension to a cryovial and immediately place cryovials in a Mr. Frosty container.
8. Transfer the container in a -80°C freezer.

CRITICAL STEP Do not keep the cryovials in the -80°C freezer for a long term.

9. Transfer the cryovials into a liquid nitrogen tank.

CAUTION

Wear protective gloves and a face mask while handling liquid nitrogen

PAUSE POINT

Cells can be frozen in liquid nitrogen for several years.

## Box

**Editorial Summary**: Protocol for the generation of human blastoids – structures morphologically and transcriptionally resembling complete blastocysts – from pluripotent stem cells and for their use in modelling implantation onto hormonally stimulated endometrial cells.

**Proposed Teaser**: Human blastoids model blastocyst-stage embryos.
