# Supplementary material for: Generating human blastoids modeling blastocyst-stage embryos and implantation
Source: Nat Protoc. Author manuscript; Available in PMC 2024 Dec 11. (PMC7617227; doi:10.1038/s41596-023-00802-1)
Supplement: Supplementary Materials [file EMS201642-supplement-Supplementary_Materials.zip › 41596_2023_802_MOESM1_ESM.pdf]

nature portfolio

https://doi.org/10.1038/s41596-023-00802-1

Supplementary information

Generating human blastoids modeling blastocyst-stage embryos and implantation

In the format provided by the authors and unedited

nature portfolio

https://doi.org/10.1038/s41596-023-00802-1

Supplementary information

Generating human blastoids modeling blastocyst-stage embryos and implantation

In the format provided by the authors and unedited

Supplementary Table 1. Different platforms for human blastoid formation

Description	Cat. No.	Microwell Size (Diameter-µm)	Recommended Working Volume	Number of Microwells/Well	Number of Microwells/plate	Recommended cell number for seeding (target: 45 ± 10 cells/microwells)	Recommended final medium volume for seeding (µl)
96 well-plates hydrogel microwell array-200	In house	200	200	400-430	around 38000	30000	200
96 well-plates hydrogel microwell array-400	in house	400	200	60-70	around 6700	6000±500	200
Corning Elplasia 96-well round bottom microplate	4442	500	200	80	around 7500	6000±500	200
Gri3D® 96 Wellplate	200 - Custom	200	200	500	around 48000	36000	200
AggreWell™400	34421	400	500-1500	1200	around 28000	90000-100000	500

Description	Changing medium for the D0	Changing medium for D1	Changing medium from D2-D4	Comments
96 well-plates hydrogel microwell array-200	Aspirate all the medium and add 200 µl of PALLY medium	Aspirate all the medium and add 200 µl of PALLY medium	Aspirate all the medium and add 200 µl of the new medium	Time/cost investment
96 well-plates hydrogel microwell array-400	Aspirate 100 µl of the medium and add 100 µl of 2X PALLY medium	Aspirate 150 µl of the medium and add 200 µl of PALLY medium	Aspirate 200 µl of the medium and add 200 µl of the new medium	Time/cost investment
Corning Elplasia 96-well round bottom microplate	Aspirate 100 µl of the medium and add 100 µl of 2X PALLY medium	Aspirate 150 µl of the medium and add 200 µl of PALLY medium	Aspirate 200 µl of the medium and add 200 µl of the new medium	Lower optical properties in brightfield
Gri3D® 96 Wellplate	Aspirate all the medium and add 200 µl of PALLY medium	Aspirate all the medium and add 200 µl of PALLY medium	Aspirate all the medium and add 200 µl of the new medium	Expensive price
AggreWell™400	Add 500 µl of the 2X PALLY medium	Aspirate 500 µl of the medium and add 500 µl of PALLY medium	Aspirate 500 µl of the medium and add 500-1000 µl of the new medium	Large number of cells required per well<br>Changing media is difficult<br>Cavitated structures begin to float<br>Not compatible for fluorescence and live imaging<br>Not compatible for imaging-based drug screening

Supplementary Table 2. Summary of patient information

Donor ID	Age	Cycle	Cycle determination	Clinical information
Donor 1	24	late proliferative phase	Last menstrual period (LMP)	ICSI for male factor infertility
Donor 2	27	Secretory phase	Last menstrual period (LMP)	ICSI for male factor infertility
Donor 3	28	Secretory phase	Last menstrual period (LMP)	ICSI for male factor infertility
