# Supplementary material for: Generating human blastoids modeling blastocyst-stage embryos and implantation
Source: Nat Protoc. Author manuscript; Available in PMC 2024 Dec 11. (PMC7617227; doi:10.1038/s41596-023-00802-1)
Supplement: Supplementary Video Legends [file EMS201642-supplement-Supplementary_Video_Legends.pdf]

Supplementary materials

Video 1. Human blastoids attach via the polar trophectoderm. Live imaging of human blastoids attached via the polar region to hormonally stimulated layers of endometrial epithelial cells, and challenged by pipetting liquid in their vicinity.

Video 2. Human blastoids on non-hormonally stimulated endometrial layers. Live imaging of human blastoids on non-hormonally stimulated layers of endometrial epithelial cells, and challenged by pipetting liquid in their vicinity. Blastoids do not show attachment.
